# Symmetry as a common origin of hierarchical emergence and abstraction

**DOI:** 10.1126/sciadv.aeh0773

**Published:** 2026-11-13

**Authors:** Fernando E. Rosas

**Affiliations:** ^1^ Sussex AI and Sussex Centre for Consciousness Science, Department of Informatics, University of Sussex, Brighton, UK.; ^2^ Centre for Complexity Science and Department of Brain Sciences, Imperial College London, London, UK.; ^3^ Centre for Eudaimonia and Human Flourishing, University of Oxford, Oxford, UK.; ^4^ XOR Labs, London, UK.

## Abstract

Many systems of interest exhibit nested emergent layers with their own rules and regularities, and our knowledge about them seems naturally organized around these levels. How do such hierarchical structures arise, and why should observers track these hierarchies so closely? This paper proposes symmetry as an answer to both questions. By combining principles from information theory, group theory, and statistical mechanics, it is shown that dynamical processes that are equivariant with respect to a symmetry group give rise to multiple emergent macroscopic levels arranged according to the algebraic structure of the symmetry. The same symmetries shape the Bayesian beliefs of an observer with only partial access to the system, yielding a matching hierarchy of “abstract” beliefs that can be updated autonomously at different levels of resolution. These ideas are illustrated in Hopfield networks, where familiar macroscopic quantities emerge from known symmetries, and in Boolean models of gene regulation, where symmetries are instead found from the dynamics. The computational advantages provided by symmetry are showcased in Ehrenfest diffusion and epidemic modeling, where abstract beliefs enable Bayesian inference over exponentially large state spaces. These results identify symmetry as a sufficient mechanism for hierarchical emergence and—at the same time—for the capacity of abstract beliefs to mirror the systems they track, making feasible inferential problems that would otherwise be intractable.

## INTRODUCTION

Our ability to successfully navigate and learn about the world, despite the limitations imposed by no free lunch theorems (*1*, *2*), suggests that our world has special properties that make this success possible. A key factor enabling this success seems to be that the world presents itself in the form of nested yet distinct layers of organization—a property often called “emergence” (*3*, *4*). Emergence gives rise to a separation of scales that allows each level to exhibit its own rules and regularities, which can be learned and exploited while disregarding the activity of levels below (*5*–*7*). This hierarchical architecture is mirrored in the organization of knowledge into scientific disciplines (physics → chemistry → biology → sociology and economics), where each field abstracts away details of the levels below to form its own effective theories (*8*).

Emergence has been studied in statistical physics via phase transitions and renormalization group theory, which provide a powerful framework that describes how separation of scales occurs and what the resulting emergent laws are (i.e., universality) (*9*–*12*). Complementing this work, a line of investigations inspired by theoretical neuroscience has focused on multiscale dynamics, characterizing various aspects of emergence in terms of information-theoretic principles (*13*–*16*). These approaches provide tools to identify when emergent levels arise in the dynamics of general systems far from the thermodynamic limit, but have not yet identified fundamental principles that can explain how this happens. Furthermore, it is currently unclear how existing approaches to study emergence could account for its ability to enhance learnability and inference.

Building on these developments, this work proposes symmetry as a driver of hierarchical emergence in both dynamical processes and epistemic processes involving beliefs about unobserved variables. The results presented here show that when the dynamics of a stochastic process commute with a symmetry group, they naturally induce coarse-grained variables whose evolution is informationally self-contained—so that microscopic details are irrelevant for answering questions regarding their past or future evolution. Moreover, the algebraic structure of the symmetry group reveals a whole hierarchy of emergent variables, where subgroups correspond to finer partitions of the microscopic state space. The same symmetries also shape the Bayesian beliefs held by an observer who only has partial access to the system, giving rise to a hierarchy of coarse-grained beliefs matching the levels of the observed process. These matching hierarchies allow the tracking of macroscopic properties of high-dimensional systems while disregarding low-level details, replacing exponentially large microscopic belief states with macroscopic beliefs that greatly simplify inference problems.

The relation between emergence and symmetry is illustrated in case studies involving paradigmatic models from statistical mechanics and computational neuroscience, where familiar macroscopic quantities (such as magnetization or particle count) emerge as self-contained macroscopic processes arising from underlying symmetries. The plausibility of recovering the hierarchical structure from dynamics is illustrated in the analysis of Boolean models of gene regulation, where the hierarchy of levels can be extracted from the transition probabilities. The computational advantages enabled by symmetries are demonstrated in epidemic modeling, allowing for exact inference over the infection count in heterogeneous populations with exponentially large state spaces.

### Background: Emergence and informational closure

Emergence is a rich subject that can be investigated from various perspectives (*3*, *4*, *17*–*19*). In this work, emergence is conceptualized as the successful factoring out of microscopic laws without loss of relevant explanatory power (*5*, *6*). Emergence allows systems to exhibit separation of scales giving rise to effective theories—new and compressed descriptions operating on derived degrees of freedom (*7*, *8*). Thus, emergent properties are not efficiently described at their microscopic levels but through coarse-graining, leading to effective high-level descriptions that provide levers for design and control (*20*–*23*).

To formalize this view, consider a system whose state is specified by the random variable $X_{t}\in X$ that is measured at discrete time points t∈ℤ. A coarse-graining mapping ϕ:X→Z gives rise to another process $Z_{t}=\phi \left(X_{t}\right)$. A coarse-graining $Z_{t}$ is said to be informationally closed if, for predicting a macroscopic future ${\vec{Z}}_{t+1}^{\;L}=\left(Z_{t+1},\ldots ,Z_{t+L}\right)$, knowing the microscopic past ${\overset{\leftarrow }{X}}_{t}=\left(\ldots ,X_{t-1},X_{t}\right)$ offers no advantage over knowing only the macroscopic past ${\overset{\leftarrow }{Z}}_{t}=\left(\ldots ,Z_{t-1},Z_{t}\right)$, implying that all relevant information has “moved up” to the coarse-grained level (*16*). This condition is captured by the following information-theoretic formulation (*24*, *25*)

$$I\left({\overset{\leftarrow }{Χ}}_{t};{\vec{Z}}_{t+1}^{\;L}\right)-I\left({\overset{\leftarrow }{Z}}_{t};{\vec{Z}}_{t+1}^{\;L}\right)=0\;\forall L\in \mathbb{N},t\in \mathbb{Z}$$ (1)

where *I* is Shannon’s mutual information. Information closure is equivalent to

$$D_{\text{KL}}\left(p\left({\vec{Z}}_{t+1}^{\;L}|{\overset{\leftarrow }{X}}_{t}\right)∥p\left({\vec{Z}}_{t+1}^{\;L}|{\overset{\leftarrow }{Z}}_{t}\right)\right)=0\;\forall L\in \mathbb{N},t\in \mathbb{Z}$$ (2)

where $D_{\text{KL}}$ is the Kullback-Leibler divergence. This condition ensures that $p\left({\vec{Z}}_{t+1}^{\;L}|{\overset{\leftarrow }{X}}_{t}\right)=p\left({\vec{Z}}_{t+1}^{\;L}|{\overset{\leftarrow }{Z}}_{t}\right)$ almost surely, which implies that the distinct past conditions of the system that lead to different future macroscopic outcomes can be effectively set from a suitable macroscopic preparation (*6*).

Information closure provides a useful foundation to conceptualize emergence, but it has important limitations. First, information closure does not guarantee that the resulting process is nontrivial [e.g., the constant coarse-graining $\phi \left(X_{t}\right)=c$ satisfies Eq. 1]. Second, while information closure guarantees that optimal prediction of future macroscopic events relies only on macroscopic information, it does not provide guarantees for the retrodiction of past macroscopic events (Corollary 1). This work proposes symmetries as a way to address both limitations: Symmetries are shown to yield a structured hierarchy of coarse-grainings that are fully informationally self-contained (i.e., both forward and backward in time), and are thus amenable to description by their own effective theories. The condition of having a hierarchy of informationally self-contained levels is henceforth described as hierarchical emergence.

## RESULTS

### Symmetry and hierarchical emergence

#### Dynamical symmetries

Consider a process $X_{t}$ taking values on a set X and a group *G* acting on X, so that there exist mappings g(⋅):X→X for all g∈G such that $\left(g_{1}g_{2}\right)\cdot x=g_{1}\cdot \left(g_{2}\cdot x\right)$. Group actions may permute interchangeable components of a system, but can also be more general. A group action induces a partition on X via orbits

$$O_{x}≔\left\{x^{\prime }\in X:g\cdot x=x^{\prime },g\in G\right\}$$ (3)

which leads to the coarse-graining $Z_{t}=\phi_{G}\left(X_{t}\right)$ given by $\phi_{G}\left(x\right)≔O_{x}$. For simplicity, the presentation will focus on finite groups *G* and discrete random variables.

Under what conditions may the orbits of *G* generate an informationally closed level? To investigate how the dynamics interact with the symmetry, one can use the twirling operator (*26*–*28*)

$$R\left\{\mu \right\}=\frac{1}{|G|}\sum_{g\in G}\mu \circ g$$ (4)

which corresponds to averaging all symmetry-equivalent versions of the dynamics. When applied on the conditional law over future blocks $K_{t+1}^{L}\left({\overset{\leftarrow }{x}}_{t}\right)≔p\left({\vec{x}}_{t+1}^{\;L}|{\overset{\leftarrow }{x}}_{t}\right)$, the twirling operator gives

$$R\left\{K_{t+1}^{L}\right\}\left({\overset{\leftarrow }{x}}_{t}\right)=\frac{1}{{|G|}^{L}}\sum_{{\vec{g}}_{t+1}^{\;L}\in G^{L}}p\left({\vec{g}}_{t+1}^{\;L}\cdot {\vec{x}}_{t+1}^{\;L}|{\overset{\leftarrow }{x}}_{t}\right)$$ (5)

where ${\vec{g}}_{t+1}^{\;L}\cdot {\vec{x}}_{t+1}^{\;L}=\left(g_{t+1}\cdot x_{t+1},\ldots ,g_{t+L}\cdot x_{t+L}\right)$ is the component-wise application of a trajectory of group elements, and $G^{L}$ is the *L*th Cartesian product of *G*. Intuitively, the dynamics respect the symmetry if the result of applying the twirling operator to the conditional law is invariant to the group action. The next result shows that such invariance is equivalent to the symmetry orbits yielding an informationally closed coarse-graining (proof in section S1).

Theorem 1. *The coarse-graining* $Z_{t}=\phi_{G}\left(X_{t}\right)$ *is informationally closed if and only if* $R\left\{K_{t+1}^{L}\right\}\left({\overset{\leftarrow }{x}}_{t}\right)$ *is invariant to the component-wise action of G for all* L∈ℕ*,* t∈ℤ*.*

#### Markov processes and generalized symmetries

Theorem 1 has important consequences for Markov processes. To unpack these, let us first distinguish three different types of symmetries that can take place in Markov chains.

Global symmetries correspond to automorphisms of the process’ transition matrix seen as a weighted directed graph. Here, states that are coarse-grained together have isomorphic incoming and outgoing transitions, and such isomorphisms compose (i.e., if $x,x^{\prime }$ are isomorphic via *f* and $x^{\prime },\hat{x}$ are isomorphic via $f^{\prime }$, then *x* and $\hat{x}$ are isomorphic via $f^{\prime }\circ f$). Global symmetries in a Markov chain $X_{t}$ correspond to dynamical equivariance: There exists a group action that satisfies

$$p\left(g\cdot x_{t+1}|g\cdot x_{t}\right)=p\left(x_{t+1}|x_{t}\right),\;\forall x_{t},x_{t+1}\in X,g\in G$$ (6)

An example of a Markov chain with a global symmetry is given in Fig. 1A. When considering deterministic transitions, Eq. 6 reduces to equivariant maps (*29*, *30*).

**Fig. 1.**
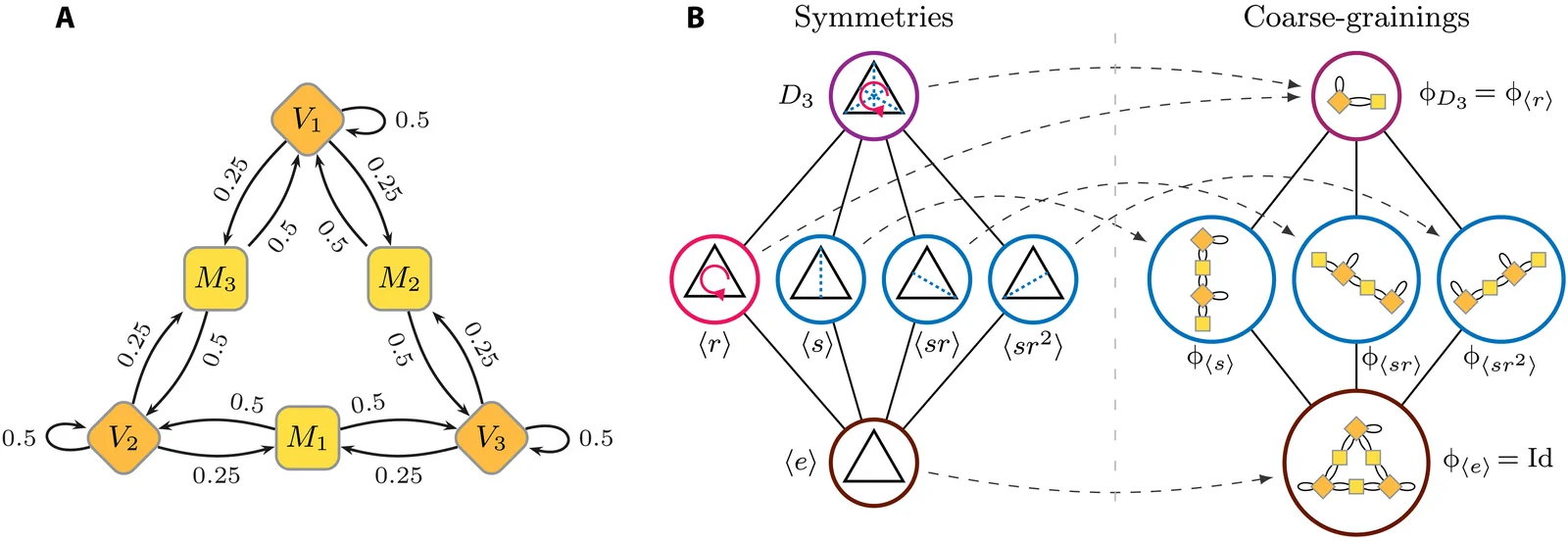
Symmetry gives rise to simpler self-contained levels. (**A**) A Markov chain over six states whose dynamics respects symmetries equivalent to those of a triangle—known as the dihedral group $D_{3}$. (**B**) Correspondence between the subgroups of $D_{3}$ and the hierarchy of self-contained coarse-grainings of the Markov chain, which preserves the partial ordering.

In contrast, local symmetries correspond to partitions C that identify states whose sets of outgoing probabilities to cells of the partition are isomorphic (though these isomorphisms need not compose). Thus, if $x,x^{\prime }$ lie in the same cell A∈C, then for every target cell B∈C, there is a bijection $\sigma_{x,x^{\prime }}^{B}:B\to B$ such that the following holds for all $\tilde{x}\in B$

$$\mathbb{P}\left\{X_{t+1}=\tilde{x}|X_{t}=x\right\}=\mathbb{P}\left\{X_{t+1}=\sigma_{x,x^{\prime }}^{B}\left(\tilde{x}\right)|X_{t}=x^{\prime }\right\}$$ (7)

This corresponds to partitions whose algebraic properties are described by groupoids, where composition is only partially defined [(*31*), section 7].

Last, weak symmetries identify states whose sets of outgoing probabilities to partition cells are isomorphic at a coarser level: They may have different weights but their sum is the same. This corresponds to a lumpable partition (*32*), which identifies $x_{t}$ and $x_{t}^{\prime }$ if

$$\mu_{\phi }\left(z_{t+1}|x_{t}\right)=\mu_{\phi }\left(z_{t+1}|x_{t}^{\prime }\right),\;\forall z_{t+1}\in Z$$ (8)

where $\mu_{\phi }\left(z_{t+1}|x_{t}\right)=\sum_{x_{t+1}\in \phi^{-1}\left(z_{t+1}\right)}p\left(x_{t+1}|x_{t}\right)$.

Global symmetries are special cases of local symmetries, which in turn are special cases of weak symmetries. The implications of each type of symmetry are outlined by the next result (proof in section S2).

Proposition 1. *Consider states of a Markov process identified under a symmetry.*

1) *A global symmetry guarantees that their trees of past and future trajectories are isomorphic.*

2) *A local symmetry guarantees their trees of future trajectories are isomorphic.*

3) *A weak symmetry guarantees their trees of future coarse-grained trajectories are isomorphic*.

Proposition 1 clarifies the guarantees provided by different symmetries, which are summarized in Table 1. Let us now consider the partition associated to a (global, local, or weak) symmetry. This partition can be used to define a macroscopic variable $Z_{t}=\phi \left(X_{t}\right)$ that tracks in which equivalence class the system is. The next result presents properties of such coarse-grainings implied by Theorem 1 and Proposition 1 (proof in section S3).

Corollary 1. *Given a Markov process* $X_{t}$*, a coarse-graining* $Z_{t}=\phi \left(X_{t}\right)$ *induced by a weak symmetry is informationally closed and Markovian. The converse holds if there is some* $t_{0}$ *with* $\mathbb{P}\left\{X_{t_{0}}=x\right\}>0$ *for all* x∈X*. If* $Z_{t}$ *is induced by a global symmetry, then it is also informationally closed under time reversal if the process has invariant marginal distributions.*

**Table 1.** Properties of various kinds of symmetry.

Kind	Algebraic form	Equivalence
Global	Group	Past and future trajectories
Local	Groupoid	Future trajectories
Weak	None	Future coarse-grained trajectories

Corollary 1 implies that symmetries of Markov processes always generate coarse-grainings that are informationally closed and Markovian. Furthermore, it highlights an important limitation: while information closure ensures that the prediction of future macroscopic states can be done optimally with macroscopic information, optimal retrodiction of past macroscopic states may need future microscopic information. Thus, information closure does not guarantee self-contained retrodiction. Global symmetries allow bidirectional information closure when having invariant marginals—which is the case, for instance, in stationary conditions for finite irreducible Markov chains.

Emergent macroscopic levels associated to a proper separation of scales should be capable of optimal self-prediction and self-retrodiction. Thus, Corollary 1 implies that coarse-grainings arising from global symmetries are better suited to characterize hierarchical emergence than lumpable partitions associated to weak symmetries. That said, conceptualizations of emergence not focused on scale separation may characterize emergent levels via different coarse-graining properties.

#### Hierarchical organization under global symmetries

So far, we have seen how a single (global, local, weak) symmetry yields one informationally closed macroscopic description. The next result shows that global symmetries bring more than that: Owing to their algebraic structure (a group rather than a groupoid), the subgroups of the global symmetry group introduce a whole hierarchy of informationally closed levels (see proof in section S4).

Corollary 2. *Let* $X_{t}$ *be a Markov process that is equivariant with respect to G, and H be a subgroup of G. If* $Z_{t}=\phi_{G}\left(X_{t}\right)$ *and* $Z_{t}^{\prime }=\phi_{H}\left(X_{t}\right)$*, then* $Z_{t}$ *is informationally closed with respect to both* $X_{t}$ *and* $Z_{t}^{\prime }$*.*

Corollary 2 implies that an equivariant Markov process gives rise to a whole hierarchy of self-contained macroscopic levels whose organization reflects the lattice structure of the subgroups of *G* (see Fig. 1B). If H⊆G, then the orbits under *G* are unions of orbits under *H*, so $Z_{t}=\phi_{G}\left(X_{t}\right)$ is coarser than $Z_{t}^{\prime }=\phi_{H}\left(X_{t}\right)$. Thus, there is a map between the lattice of subgroups of *G* and informationally self-contained coarse-grainings that preserves the partial ordering (see Fig. 1B). Within this hierarchy, the coarsest level corresponds to the group of all symmetries (*G*), and the finest level corresponds to the trivial subgroup with single-element orbits (〈e〉).

### Case study 1: Hopfield networks

Let us illustrate the relationship between hierarchical emergence and symmetry in Hopfield networks, a paradigmatic model of memory storage and retrieval (*33*–*37*). A Hopfield network is a recurrent neural network where the connection weights are designed so that certain patterns become attractors of the neural dynamics (*38*, *39*) (see Fig. 2A). The state of the *i*th neuron $X_{t}^{i}\in \left\{-1,1\right\}$ updates according to

$$\mathbb{P}\left\{X_{t+1}^{i}=1|X_{t}^{1},\ldots ,X_{t}^{n}\right\}=F\left(\beta \sum_{j=1}^{n}w_{i,j}X_{t}^{j}\right)$$ (9)

where F(x)=(1+tanhx)/2 is the activation function, $w_{i,j}$ describes the strength of the synapse from the *j*th neuron, and β regulates the stochasticity of the updates. The dynamics of the whole network $X_{t}=\left(X_{t}^{1},\ldots ,X_{t}^{n}\right)$ are given by $p\left(x_{t+1}|x_{t}\right)=\prod_{j}p\left(x_{t+1}^{j}|x_{t}\right)$.

**Fig. 2.**
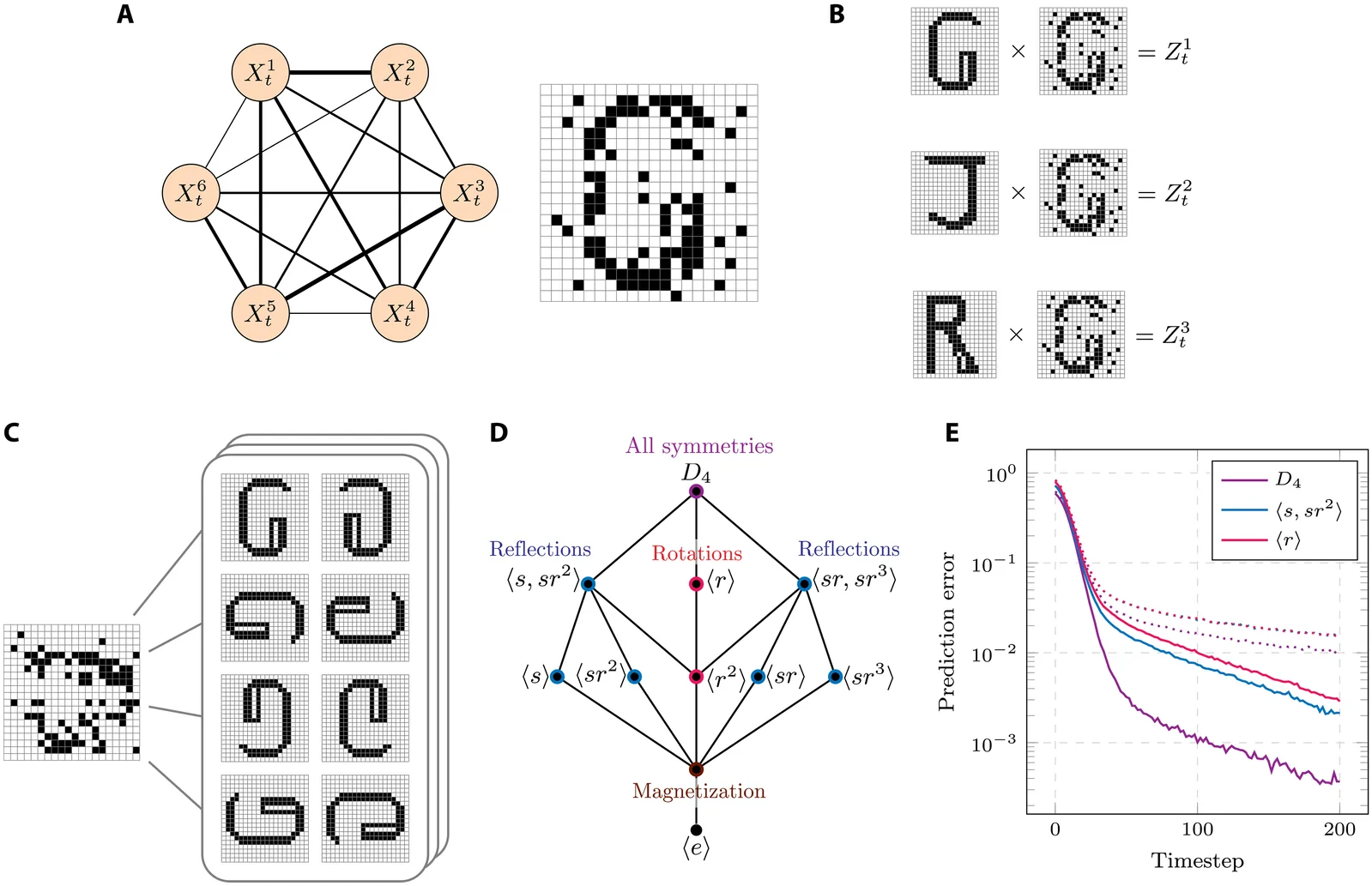
Equivariant symmetries in a Hopfield network. (**A**) A Hopfield network is a recurrent neural network trained to retrieve a given set of patterns. (**B**) The dot products between each candidate pattern and the current state of the network label the orbits of a symmetry group of the dynamics, providing natural order parameters known as Mattis magnetizations. (**C**) Training over letters and their rotations and reflections induces additional equivariances following the dihedral group $D_{4}$. (**D**) The subgroups of $D_{4}$ yield different informationally self-contained coarse-grainings. Factoring all symmetries out captures letter identity, while factoring by the rotation subgroup 〈r〉 preserves letter identity and whether it is reflected. (**E**) Experiments show that these symmetry-derived coarse-grainings (solid lines) yield better self-prediction than arbitrary coarse-grainings of the same size (dotted lines matched by color).

To store and retrieve patterns, Hopfield networks typically use Hebbian learning (*40*), which strengthens the connection between neurons that tend to coactivate. Given *m* patterns $q_{\mu }=\left(q_{\mu }^{1},\ldots ,q_{\mu }^{n}\right)\in {\left\{-1,1\right\}}^{n}$, this suggests establishing synaptic weights according to

$$w_{i,j}=\frac{1}{n}\sum_{\mu =1}^{m}q_{\mu }^{i}q_{\mu }^{j}$$ (10)

These weights make each pattern $q_{\mu }$ an attractor-like state of the dynamics (for large *n*), so the network naturally relaxes toward one of these attractors [(*39*), chapter 17.2]. Under these weights, the input potential to the *i*th neuron can be expressed as

$$\sum_{j=1}^{n}w_{i,j}X_{t}^{j}=\frac{1}{n}\sum_{\mu =1}^{m}q_{\mu }^{i}\sum_{j=1}^{n}q_{\mu }^{j}X_{t}^{j}$$ (11)

making the so-called Mattis magnetization (Fig. 2B)

$$Z_{t}^{\mu }≔\frac{1}{n}\left(q_{\mu }\cdot X_{t}\right)=\frac{1}{n}\sum_{j=1}^{n}q_{\mu }^{j}X_{t}^{j}$$ (12)

a natural order parameter of the dynamics [(*38*), chapter 4.4; see also (*41*, *42*)]. The vector of Mattis magnetizations $Z_{t}=\left(Z_{t}^{1},\ldots ,Z_{t}^{m}\right)$ tracks the degree of similarity of the current pattern $X_{t}$ with each of the candidate patterns—a typical run starts with all magnetizations being low and ends with one dominating over the rest.

To investigate the symmetries of Hopfield networks, let us build a group *G* containing all the automorphisms $\sigma :{\left\{-1,1\right\}}^{n}\to {\left\{-1,1\right\}}^{n}$ that leave the magnetization unchanged, so that σ⋅x satisfies

$$\left[q_{\mu }\cdot \left(\sigma \cdot x\right)\right]=\left(q_{\mu }\cdot x\right)\;\forall x\in {\left\{-1,1\right\}}^{n},\mu =1,\ldots ,m$$ (13)

Given that the transitions factorize as $p\left(x_{t+1}|x_{t}\right)=\kappa \left(z\left(x_{t+1}\right),z\left(x_{t}\right)\right)$, where the notation z(x) makes the dependence of magnetization on state (Eq. 12) explicit, one can show that (see section S5)

$$p\left(g\cdot x_{t+1}|g\cdot x_{t}\right)=\kappa \left(z\left(g\cdot x_{t+1}\right),z\left(g\cdot x_{t}\right)\right)=p\left(x_{t+1}|x_{t}\right)$$ (14)

so $X_{t}$ is dynamically equivariant. The Mattis magnetization corresponds to the orbits of this symmetry; thus, $Z_{t}=\phi_{G}\left(X_{t}\right)$. Because of Corollary 1, this implies that $Z_{t}$ is informationally self-contained, confirming the role of the magnetization as a macroscopic variable. This means that the process of selecting which pattern to retrieve takes place at a macroscopic level ($Z_{t}$), being (conditionally) independent of its instantiation at the level of individual neurons ($X_{t}$). This implies, for instance, that the identity of the pattern to be retrieved (specified by $Z_{t}$ for t≫1) depends on the initial magnetization $Z_{0}$ and not on the additional information provided by $X_{0}$.

To further study the effect of symmetries on Hopfield networks, let us consider a square arrangement of neurons encoding all reflections and 90° rotations of $k_{0}$ asymmetric letters, leading to $m=8k_{0}$ patterns (see Fig. 2C). This choice of patterns causes the synaptic weights, and thus also the neural dynamics (due to Eq. 9), to have additional symmetries corresponding to the dihedral group $D_{4}$—which is generated by the 90° rotation (*r*) and the horizontal reflection (*s*). Note that these symmetries arise from the training procedure (specifically, from the choice of data), being distinct from symmetries arising from the intrinsic dynamics of the system.

The resulting coarse-graining $Z_{t}^{\prime }=\phi_{D_{4}}\left(Z_{t}\right)$ corresponds to the evidence for each letter irrespective of its rotation or reflection. In addition, $D_{4}$ provides eight nontrivial proper subgroups (see Fig. 2D): three subgroups of order 4 and five subgroups of order 2. One of the groups of order 4 is cyclic generated by *r* corresponding to the rotations, and the other two groups are generated by reflections isomorphic to Klein’s group ${\mathbb{Z}}_{2}\times {\mathbb{Z}}_{2}$. The five groups of order 2 are generated by $r^{2}$, *s*, *sr*, ${\mathrm{sr}}^{2}$, and ${\mathrm{sr}}^{3}$. Each subgroup generates a distinct informationally self-contained coarse-graining: For instance, the cyclic group of order 4, 〈r〉, factors out rotational information, while the two subgroups isomorphic to Klein’s group, $\langle s,{\mathrm{sr}}^{2}\rangle$ and $\langle \mathrm{sr},{\mathrm{sr}}^{3}\rangle$, factor out information about certain reflections. Hence, the three subgroups of order 4 disentangle rotations and reflections, illuminating the compositional structure of the corresponding operations.

The theory states that the information provided at each of these levels is optimal for predicting itself in the future—e.g., the information given by $\phi_{D_{4}}\left(Z_{t}\right)$ should be sufficient to optimally predict what letter will be retrieved, irrespective of its rotation or reflection. To further explore this, Hopfield networks of 20 × 20 neurons were trained on the rotations and reflections of the letters *G*, *J*, and *R*, and $\phi_{H}\left(Z_{t}\right)$ was calculated for different subgroups *H* at various time points to predict its own value at the end of a 250-step run (see section S6 for details). Results show that the ability of informationally self-contained levels to predict their own future is substantially higher than that of randomly selected coarse-grainings of the same cardinality, which typically leak information into the micro-level (see Fig. 2E). This confirms that emergent variables derived from symmetries are not only conceptually natural but also practically desirable for prediction.

### Discovering emergent levels from dynamics

While the previous example illustrates how known symmetries can be used to better understand dynamics, one may wonder whether dynamics can be used to discover symmetries when they are not known. To study this, consider a stationary Markov process $X_{t}\in X$ and denote by *M* the ∣X∣×∣X∣ matrix of transition probabilities with components $p\left(x_{t+1}|x_{t}\right)$. The identification of dynamical equivariances of $X_{t}$ is equivalent to finding permutation matrices $P\in P_{|X|}$ satisfying ${\mathrm{PMP}}^{-1}=M$. Thus, the symmetries of $X_{t}$ correspond to

$$\text{Aut}\left(M\right)=\left\{P\in P_{|X|}:\mathrm{PM}=\mathrm{MP}\right\}$$ (15)

While testing each permutation individually is usually computationally infeasible, Aut(*M*) can often be calculated by encoding *M* as an edge-colored directed graph and exploiting its algebraic properties (see Methods).

As an example of how these ideas can be used, let us analyze the dynamics that arise from a gene-regulatory network whose state is given by $X_{t}=\left(X_{t}^{1},\ldots ,X_{t}^{n}\right)\in {\left\{0,1\right\}}^{n}$, with each component recording whether a regulatory factor is active or inactive at time *t* (*43*, *44*). The regulatory dynamics are specified by a Boolean update map $F:{\left\{0,1\right\}}^{n}\to {\left\{0,1\right\}}^{n}$ usually defined via logical rules, threshold functions, or other model-specific regulatory mechanisms (*45*, *46*). By adding stochasticity to the modeling (*47*), the network gives rise to dynamics given by

$$p\left(x_{t+1}=x^{\prime }|x_{t}=x\right)={\left(1-\epsilon \right)}^{n-d\left(x^{\prime },\;F\left(x\right)\right)}\epsilon^{d\left(x^{\prime },\;F\left(x\right)\right)}$$ (16)

where $d\left(x,x^{\prime }\right)$ is the Hamming distance between $x,x^{\prime }$, and ϵ∈[0,1] determines the noise level.

To illustrate how symmetries can be derived from such a model, we calculated the symmetries of the Boolean model of the *Escherichia coli* l-arabinose operon reported in (*48*). The state is given by *n* = 9 factors

$$X_{t}=\left(A_{t},A_{t}^{m},A_{t}^{+},C_{t},E_{t},L_{t},{\left(\mathrm{MS}\right)}_{t},{\left(\mathrm{MT}\right)}_{t},T_{t}\right)$$ (17)

where *A* and *A*^m^ denote high and medium intracellular arabinose, *A*^+^ indicates arabinose availability, *C* represents the catabolite activation state, *E* and *L* represent expression and looping states, and *MS*, *MT*, and *T* encode downstream metabolic and transport variables (see Fig. 3A). We calculated how the symmetries depend on the availability of extracellular arabinose ($a_{e}\in \left\{\text{low},\text{med},\text{high}\right\}$) and extracellular glucose ($g_{e}\in \left\{0,1\right\}$), while assuming that unbound protein regulation is active ($a^{-}=1$). For more details, see Methods.

**Fig. 3.**
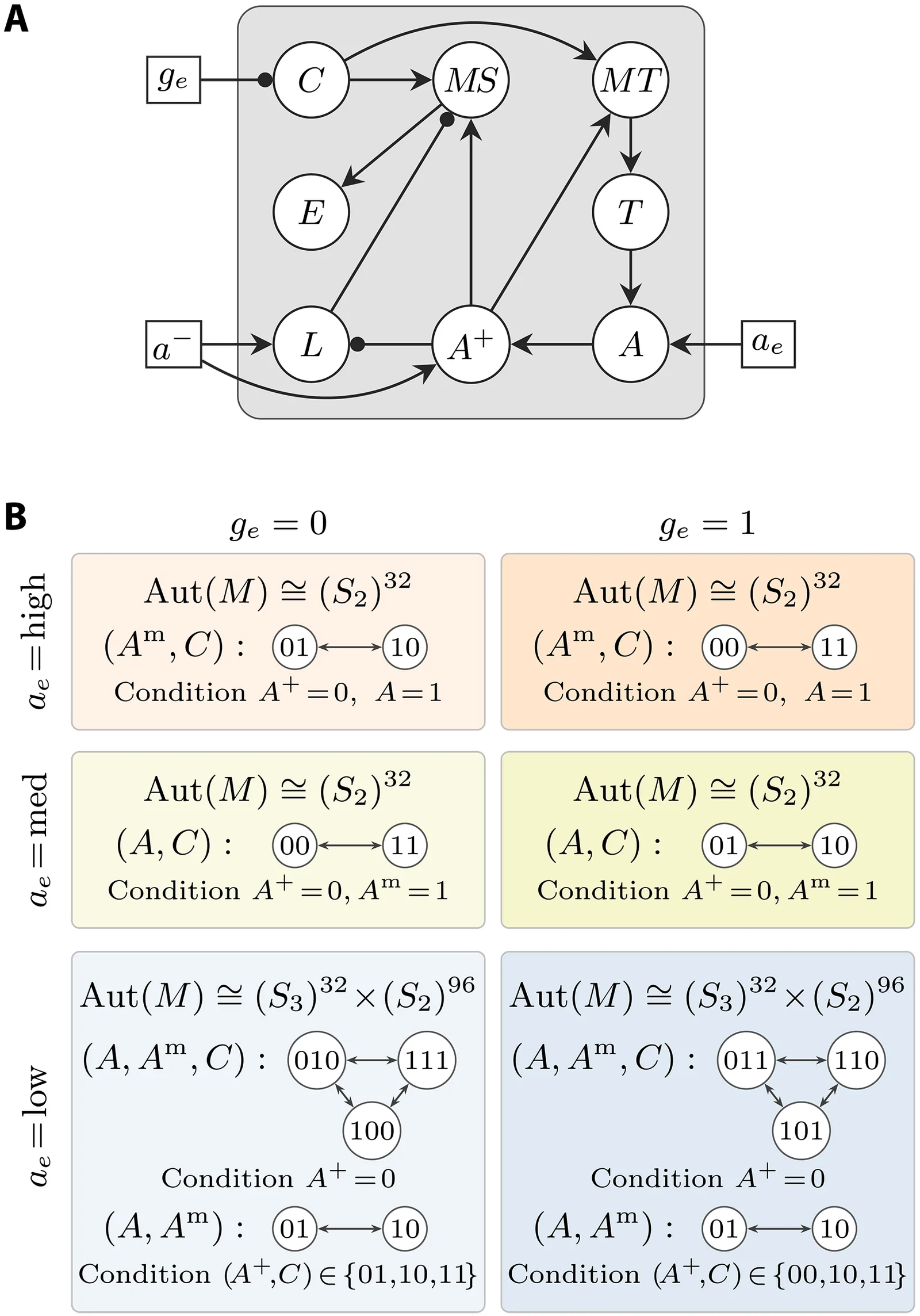
Symmetries from the dynamics of the l-arabinose operon model. (**A**) Boolean regulatory network, with nodes for the state variables and the three external inputs, and arrows indicating the regulatory interactions defined by the update map. (**B**) Symmetries found under various conditions of extracellular arabinose ($a_{e}$) and extracellular glucose ($g_{e}$), while assuming that unbound protein regulation is active ($a^{-}=1$). Overall, low extracellular arabinose makes the high (A) and medium ($A^{m}$) intracellular states interchangeable, while glucose reverses which catabolite-activation (C) configurations are grouped.

Results show that the symmetries of this system capture relational regulatory features (see Fig. 3B). Specifically, low extracellular arabinose ($a_{e}$) makes high (*A*) and medium (*A*^m^) intracellular arabinose states dynamically interchangeable, with the catabolite activation state (*C*) extending this in inactive-*A*^+^ contexts. The presence of glucose keeps the symmetries but changes which *C* configurations are grouped. For medium and high extracellular arabinose, the symmetry reduces to identifying a pair of states that differ jointly in an intracellular arabinose variable and the catabolite activation state. As before, the presence of glucose reverses the preserved relation.

### Hierarchical abstractions

After focusing on the objective dynamics of a system, we now turn to an observer who only has access to (partial and/or noisy) measurements and maintains probabilistic beliefs about its latent state. The aim is to show that the same symmetries that generate emergent macrostates give rise to an analogous hierarchy of belief states, which we describe as abstractions.

#### Multilevel latent models

Let us study the relationship between the hierarchical organization of a process and measurements of it by analyzing hidden Markov models (HMMs). In an HMM, a latent (i.e., unobservable) Markov process $X_{t}$ evolves according to a transition kernel $p\left(x_{t+1}|x_{t}\right)$, while observations $Y_{t}$ are generated through an emission kernel $p\left(y_{t}|x_{t}\right)$. This yields a joint distribution of latent states and measurements of the form

$$p\left({\vec{x}}_{0}^{\;L+1},{\vec{y}}_{0}^{\;L}\right)=p\left(x_{0}\right)\prod_{\tau =0}^{L-1}p\left(x_{\tau +1}|x_{\tau }\right)p\left(y_{\tau }|x_{\tau }\right)$$ (18)

Consider now a scenario where (i) the hidden process is dynamically equivariant and (ii) the measurements respect this symmetry. This requires two group actions from the same group, one over X and another over Y, satisfying the following conditions

$$p\left(g\cdot x_{t+1}|g\cdot x_{t}\right)=p\left(x_{t+1}|x_{t}\right)$$ (19A)

$$p\left(g\cdot y_{t}|g\cdot x_{t}\right)=p\left(y_{t}|x_{t}\right)$$ (19B)

Systems satisfying these properties will be called equivariant HMMs. The group actions can be used to build coarse-grained variables

$$Z_{t}=\phi_{G}\left(X_{t}\right)\;\text{and}\;V_{t}=\psi_{G}\left(Y_{t}\right)$$ (20)

associated with the orbits arising from these symmetries, which form themselves another HMM (proof in section S7).

Theorem 2. *If* $\left(X_{t},Y_{t}\right)$ *is an equivariant HMM, then* $\left(Z_{t},V_{t}\right)$ *is also an HMM.*

Theorem 2 shows that, if the underlying physical process and the observation are equivariant, then corresponding coarse-grainings give rise to a new HMM nested inside the original one. Furthermore, $\left(Z_{t}^{\prime },V_{t}^{\prime }\right)$ with $Z_{t}^{\prime }=\phi_{H}\left(X_{t}\right)$ and $V_{t}^{\prime }=\psi_{H}\left(Y_{t}\right)$ is also an HMM for all subgroups *H* of *G*. Thus, the algebraic properties of equivariant HMMs give rise to a whole hierarchy of simpler HMMs arranged according to the lattice structure of their symmetries. An elementary example of this is shown in Fig. 4. This allows observers to operate at multiple emergent levels, as discussed next.

**Fig. 4.**
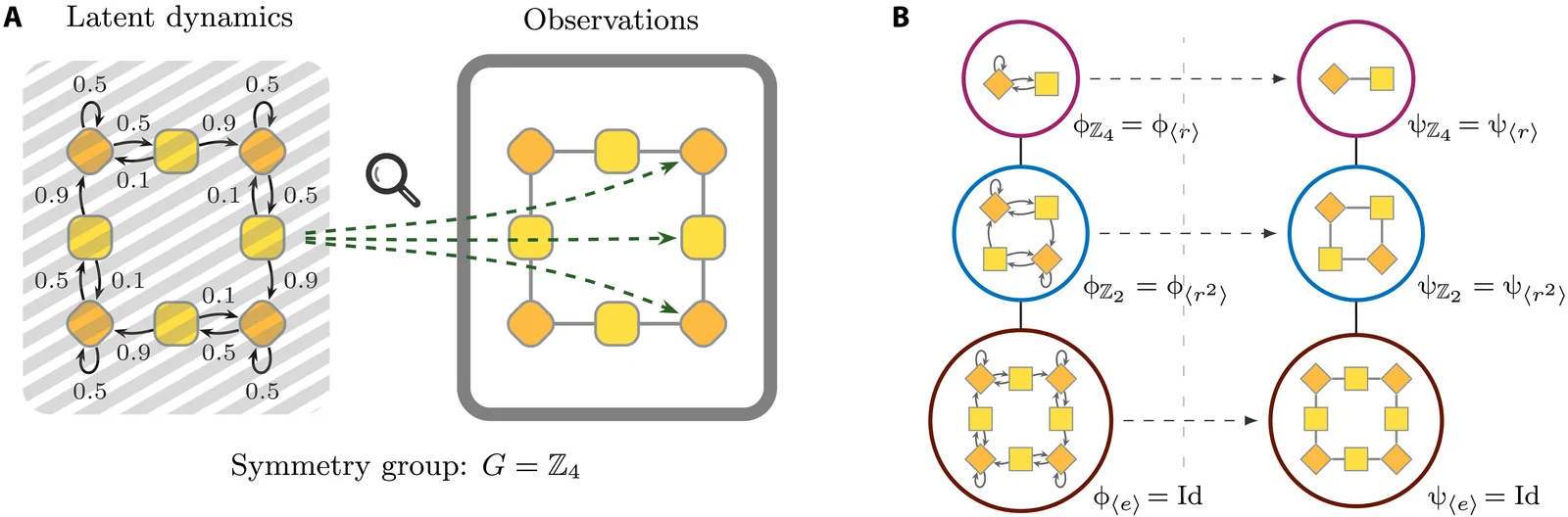
Correspondence between the hierarchy of processes and observations. (**A**) Unobserved dynamics of a Markov chain over eight states, which respects rotational symmetry as given by ${\mathbb{Z}}_{4}$. (**B**) Correspondence between the hierarchy of emergent levels of the latent dynamics (left) and levels of resolution of the observations (right). At each level, the corresponding pair $\left(\phi_{H},\psi_{H}\right)$ constitutes a valid HMM.

It is also possible to define HMMs with weak symmetries, which have more limited properties than equivariant HMMs (see Methods).

#### Hierarchical belief updating

HMMs allow us to study how (noisy or partial) measurements inform us about latent processes. One approach to this is by estimating $X_{t}$ based on a sequence of measurements ${\overset{\leftarrow }{Y}}_{t}$ using Bayesian beliefs (*49*): a Markov process $B_{t}≔E\left\{1_{X_{t}}|{\overset{\leftarrow }{Y}}_{t}\right\}$ where $1_{X_{t}}\;:=1\left\{X_{t}=x_{t}\right\}$ is a one-hot encoding of $X_{t}$. When conditioned on a realization $\left\{{\overset{\leftarrow }{Y}}_{t}={\overset{\leftarrow }{y}}_{t}\right\}$, Bayesian beliefs take the form $b_{t}\in {\mathbb{R}}^{|X|}$ with $b_{t}\left(x_{t}\right)=p\left(x_{t}|{\overset{\leftarrow }{y}}_{t}\right)$, which can be efficiently updated via Bayesian filtering as (*49*)

$$b_{t}=\text{Norm}\left(\text{diag}\left(L_{y_{t}}\right)T_{t}b_{t-1}\right)≔U_{x,y}\left(y_{t},b_{t-1}\right)$$ (21)

Above, $T_{t}\in {\mathbb{R}}^{|X|\times |X|}$ is a transition probability matrix with components ${\left[T_{t}\right]}_{x_{t},x_{t-1}}=p\left(x_{t}|x_{t-1}\right)$, $L_{y_{t}}$ is a ∣X∣-dimensional likelihood vector with components ${\left[L_{y_{t}}\right]}_{x_{t}}=p\left(y_{t}|x_{t}\right)$, and $\text{Norm}\left(v\right)=v/\sum_{j}v_{j}$. Intuitively, $T_{t}$ propagates the belief through the dynamics and then $\text{diag}\left(L_{y_{t}}\right)$ reweights the result according to the evidence brought by $y_{t}$.

It is possible to build beliefs about arbitrary coarse-grained versions of the latent process $W_{t}=f\left(X_{t}\right)$, resulting in $B_{t}^{f}≔E\left\{1_{W_{t}}|{\overset{\leftarrow }{y}}_{t}\right\}$ and $b_{t}^{f}≔p\left(w_{t}|{\overset{\leftarrow }{y}}_{t}\right)$. These beliefs satisfy $B_{t}^{f}=P_{f}B_{t}$, where $P_{f}\in {\left\{0,1\right\}}^{|W|\times |X|}$ is a matrix with components ${\left[P_{f}\right]}_{w,x}=1\left\{w=f\left(x\right)\right\}$. However, most such coarse-grainings result in $B_{t}^{f}$ being non-Markovian, which forbids the use of update mechanisms analogous to Eq. 21. Coarse-grainings associated with equivariant symmetries circumvent these issues, resulting in Markovian beliefs that can be updated efficiently (see proof in section S8).

Corollary 3. *The beliefs* $b_{t}^{*}≔p\left(z_{t}|{\overset{\leftarrow }{v}}_{t}\right)$ *of an equivariant HMM can be updated* via $b_{t}^{*}=U_{z,v}\left(v_{t},b_{t-1}^{*}\right)$*, with U as defined in* *Eq. 21**. Moreover,* $b_{t}^{*}$ *are related to the micro-level beliefs via*

$$b_{t}^{*}=E\left\{P_{\phi_{G}}B_{t}|{\overset{\leftarrow }{V}}_{t}={\overset{\leftarrow }{v}}_{t}\right\}$$ (22)

Thus, just as symmetries induce emergent macrostates in the physical system, Corollary 3 reveals they also induce simpler belief states—henceforth called abstract beliefs—that can be used for doing inference over self-contained levels of the latent process (see Fig. 5). These abstract beliefs are built via Eq. 22, which mixes micro-level beliefs corresponding to measurement sequences that differ only by symmetry transformations

$$\Psi_{G}\left\{b_{t}\left[{\overset{\leftarrow }{y}}_{t}\right]\right\}≔E\left\{1_{Z_{t}}|{\overset{\leftarrow }{V}}_{t}=\psi_{G}\left({\overset{\leftarrow }{y}}_{t}\right)\right\}$$ (23)

$$=P_{\phi_{G}}\left(\sum_{{\overset{\leftarrow }{y}}_{t}^{\prime }:\psi_{G}\left({\overset{\leftarrow }{y}}_{t}^{\prime }\right)=\psi_{G}\left({\overset{\leftarrow }{y}}_{t}\right)}c_{{\overset{\leftarrow }{y}}_{t}^{\prime }}b_{t}\left[{\overset{\leftarrow }{y}}_{t}^{\prime }\right]\right)$$ (24)

where the notation $b_{t}\left[{\overset{\leftarrow }{y}}_{t}\right]=p\left(x_{t}|{\overset{\leftarrow }{y}}_{t}\right)$ highlights the dependency of the belief on the measurement sequence ${\overset{\leftarrow }{y}}_{t}$, and $c_{{\overset{\leftarrow }{y}}_{t}^{\prime }}$ are mixing coefficients given by

$$c_{{\overset{\leftarrow }{y}}_{t}^{\prime }}=p\left({\overset{\leftarrow }{y}}_{t}^{\prime }|\psi_{G}\left({\overset{\leftarrow }{y}}_{t}\right)\right)=\frac{p\left({\overset{\leftarrow }{y}}_{t}^{\prime }\right)}{\sum_{{\overset{\leftarrow }{y}}_{t}^{\prime }:\psi_{G}\left({\overset{\leftarrow }{y}}_{t}^{\prime }\right)=\psi_{G}\left({\overset{\leftarrow }{y}}_{t}\right)}p\left({\overset{\leftarrow }{y}}_{t}^{\prime }\right)}$$ (25)

**Fig. 5.**
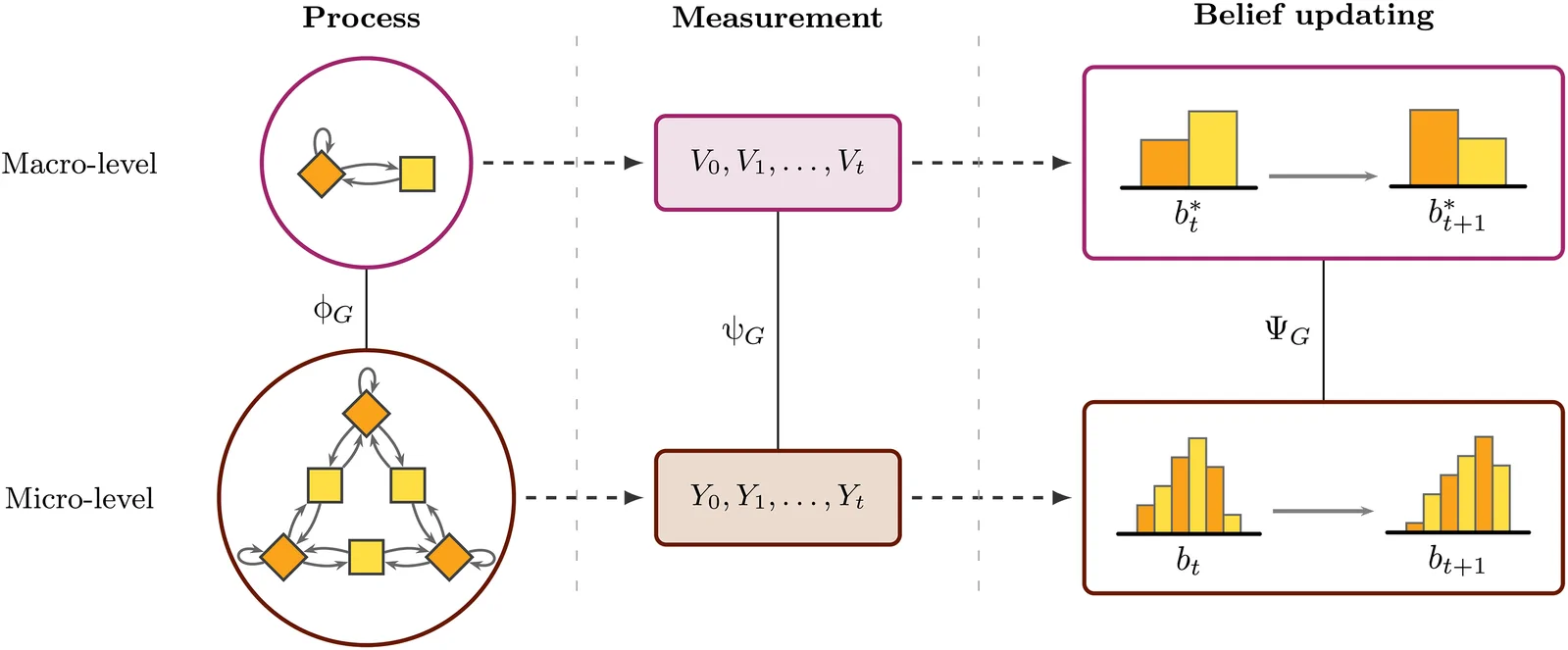
Hierarchical Bayesian belief updating. A process with equivariant symmetries has informationally self-contained levels (**left**). If measurements respect the same symmetries, then there is a correspondence between the various levels of the process and measurements of different degrees of resolution (**center**). This hierarchical structure of processes and measurements gives rise to Bayesian beliefs that can be updated autonomously at various scales, without the need to account for the information from levels below (**right**).

This mapping can be seen as a two-step process: It first mixes the micro-level beliefs $b_{t}\left[{\overset{\leftarrow }{y}}_{t}^{\prime }\right]$ in the equivalence class ${\overset{\leftarrow }{y}}_{t}^{\prime }\in \psi_{G}^{-1}\left(\psi_{G}\left({\overset{\leftarrow }{y}}_{t}\right)\right)$, and then marginalizes the mixture via $P_{\phi_{G}}$. While Eq. 24 holds for the beliefs $p\left(w_{t}|{\overset{\leftarrow }{y}}_{t}\right)$ of any coarse-graining of the latent process $W_{t}=f\left(X_{t}\right)$, these beliefs are generally non-Markovian and cannot be updated as in Eq. 21. The possibility of efficiently updating abstract beliefs by direct Bayesian filtering—without reference to the micro-level beliefs—makes these beliefs special.

While Eq. 24 establishes how to build abstract beliefs from micro-level ones, this generally does not yield a well-defined mapping between individual beliefs. A sufficient condition for such mapping to exist is given next (see proof in section S9).

Corollary 4. *Abstract beliefs of equivariant HMMs satisfy* $b_{t}^{*}=P_{\phi_{G}}b_{t}$ *if*

$$I\left(Z_{t};{\overset{\leftarrow }{Y}}_{t}\right)-I\left(Z_{t};{\overset{\leftarrow }{V}}_{t}\right)=0$$ (26)

Equation 26 states that the coarse-grained measurement ${\overset{\leftarrow }{V}}_{t}$ contains all the information about $Z_{t}$ that exists in the full measurement ${\overset{\leftarrow }{Y}}_{t}$, being analogous to information closure at the belief level. An equivalent condition is

$$p\left(z_{t}|{\overset{\leftarrow }{y}}_{t}\right)=p\left(z_{t}|g_{0}\cdot y_{0},\ldots ,g_{t}\cdot y_{t}\right),\;\forall g_{k}\in G$$ (27)

If these conditions hold, then abstract beliefs are just a marginalization of micro-level beliefs. This, in turn, ensures that the following diagram commutes:


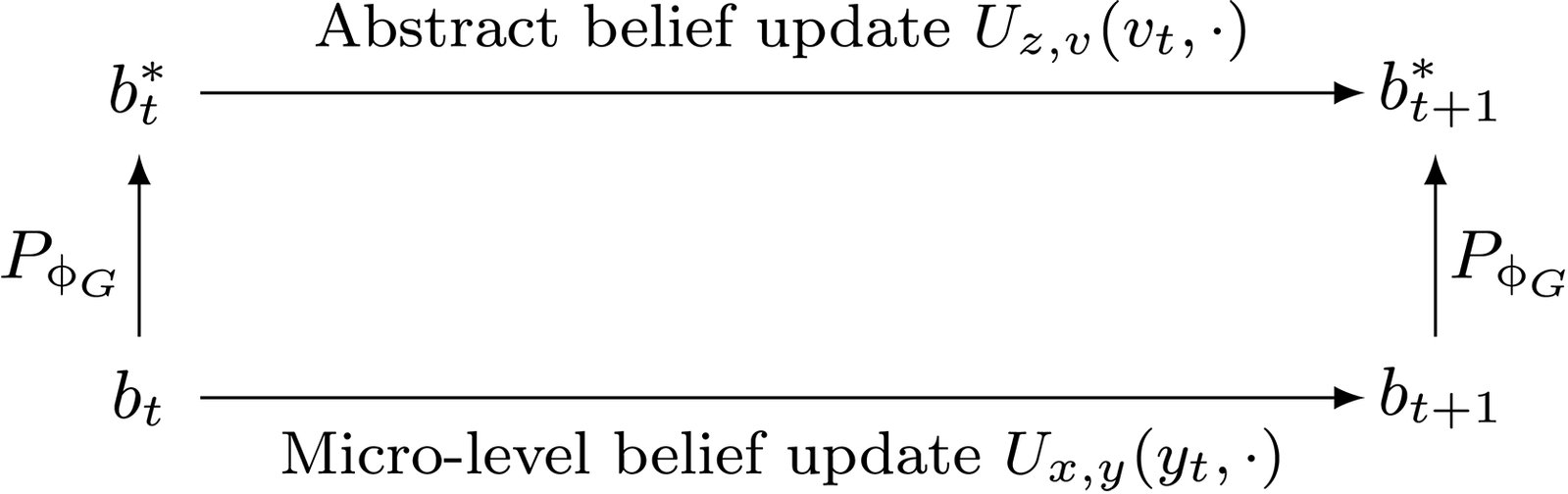


This guarantees that Bayesian updating at different levels of abstraction is fully compatible: Updating beliefs at a coarse level corresponds to marginalizing the fine-grained beliefs, and vice versa. In contrast, when Eq. 26 does not hold, then $I\left(Z_{t};{\overset{\leftarrow }{Y}}_{t}\right)>I\left(Z_{t};{\overset{\leftarrow }{V}}_{t}\right)$, meaning that the beliefs $p\left(z_{t}|{\overset{\leftarrow }{y}}_{t}\right)=P_{\phi_{G}}b_{t}$ are more informative about $Z_{t}$ than $b_{t}^{*}$. However, there may not exist an efficient way to update $p\left(z_{t}|{\overset{\leftarrow }{y}}_{t}\right)$ without updating $b_{t}$ first [as $\left(Z_{t},Y_{t}\right)$ may not be an HMM], making $b_{t}^{*}$ a less informative but more tractable alternative. Note that, because of the tower property of conditional expectations, Eq. 22 guarantees that both $b_{t}^{*}$ and $P_{\phi_{G}}b_{t}$ always agree on their mean, but their variance will generally be different.

In summary, beliefs arising from equivariant HMMs can be updated at different levels of granularity, depending on the underlying symmetries. Updating $b_{t}=p\left(x_{t}|{\overset{\leftarrow }{y}}_{t}\right)$ gives estimates of the system’s microscopic state, but their updating and storage can involve prohibitive computational costs. In contrast, updating $b_{t}^{*}=p\left(z_{t}|{\overset{\leftarrow }{v}}_{t}\right)$ gives estimates of a macroscale description of the system, resulting in lower-dimensional beliefs whose updating and storage can be substantially cheaper.

### Case study 2: Ehrenfest’s model

Let us illustrate these results on Ehrenfest’s diffusion model (*50*), a classic model from statistical mechanics that describes the behavior of gas in a container made of two interconnected chambers. We will see how permutation symmetry at the microstate gives rise to the number of particles per chamber as a natural order parameter, over which efficient inference is possible.

The model describes the state of *n* gas particles via a binary vector $X_{t}=\left(X_{t}^{1},\ldots ,X_{t}^{n}\right)$, where $X_{t}^{k}\in \left\{0,1\right\}$ determines in which of the two chambers the *k*th particle is located (see Fig. 6A). The model assumes that at most one particle may switch between chambers at each time point, giving rise to the following dynamics

$$p\left(x_{t+1}|x_{t}\right)=\left\{\begin{array}{cc} 1-q & \text{if}\;x_{t+1}=x_{t},\\ q/n & \text{if}\;d\left(x_{t+1},x_{t}\right)=1,\\ 0 & \text{otherwise ,} \end{array}\right.$$ (28)

where *q* is the probability of a particle switching and $d\left(x,x^{\prime }\right)$ is the Hamming distance between *x* and $x^{\prime }$.

**Fig. 6.**
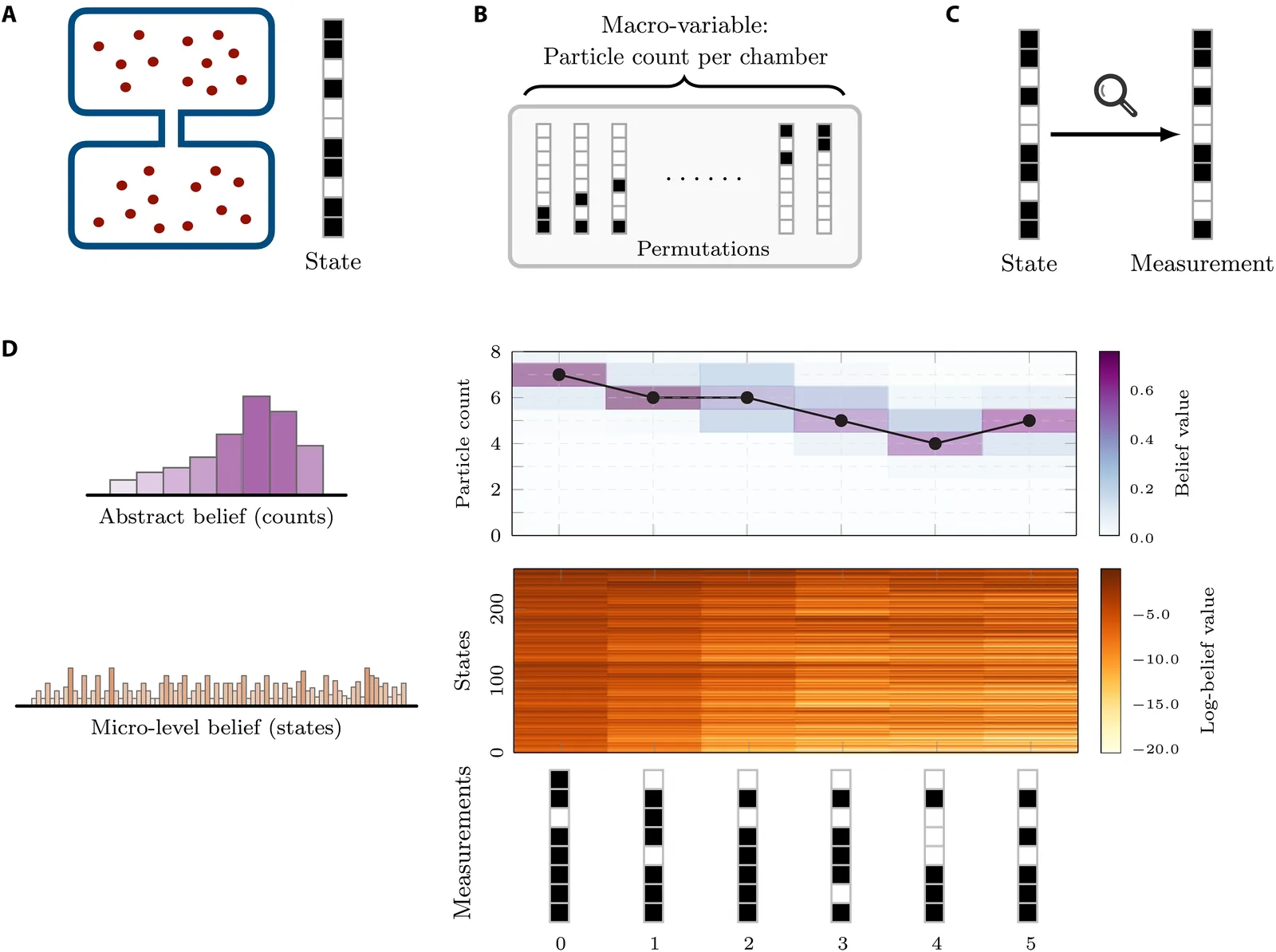
Abstract beliefs in the Ehrenfest diffusion model. (**A**) The model considers *n* particles contained in two connected chambers. The system’s state is a binary vector that specifies which chamber each particle is in. (**B**) Dynamics are equivariant under particle permutation, giving rise to orbits corresponding to the particle count per chamber. (**C**) Measurements are noisy observations of each particle’s location obtained via a binary symmetric channel with crossover probability *r*. (**D**) Updates of micro-level beliefs about the microstate and abstract beliefs about the particle count for *n* = 8, *q* = 0.9, and *r* = 0.1, both starting from a uniform prior. The dimensionality of abstract beliefs grows linearly with *n*, while the dimensionality of micro-level beliefs grows exponentially.

Consider also measurements $Y_{t}=\left(Y_{t}^{1},\ldots ,Y_{t}^{n}\right)$, where $Y_{t}^{k}\in \left\{0,1\right\}$ is an estimate of $X_{t}^{k}$ obtained via a binary symmetric channel with crossover probability *r*, so that

$$p\left(y_{t}|x_{t}\right)=\prod_{k=1}^{n}r^{1\left\{x_{t}^{k}\neq y_{t}^{k}\right\}}{\left(1-r\right)}^{1\left\{x_{t}^{k}=y_{t}^{k}\right\}}$$ (29)

From these measurements, one can build beliefs about $X_{t}$ of the form $b_{t}=p\left(x_{t}|{\overset{\leftarrow }{y}}_{t}\right)$. Unfortunately, updating and storing such beliefs is computationally infeasible for systems with more than a few particles: The dimensionality of $b_{t}$ is $|X|=2^{n}$, so the calculation of one pass of Eq. 21 requires $O\left(2^{2n}\right)$ operations.

Let us use symmetries to instead build and update beliefs about macroscopic properties. Given that gas particles are identical in this model, the dynamics of $X_{t}$ are equivariant with respect to the permutation group $S_{n}$ (see Fig. 6B). By defining

$$\sigma \cdot x_{t}≔\left(x_{t}^{\sigma \left(1\right)},\ldots ,x_{t}^{\sigma \left(n\right)}\right)\;\text{for}\;\sigma \in S_{n}$$ (30)

one can use Eq. 28 to verify that $p\left(\sigma \cdot x_{t+1}|\sigma \cdot x_{t}\right)=p\left(x_{t+1}|x_{t}\right)$ holds. The resulting orbits are given by $Z_{t}=\phi_{S_{n}}\left(X_{t}\right)=\sum_{k=1}^{n}X_{t}^{k}$, which keeps track of the dynamics of the number of particles in each chamber. Given the dynamics of the Ehrenfest model, $Z_{t}$ can be seen to follow a Markovian birth-death process—because only one particle can flip per time step, so $Z_{t}$ increases or decreases by one with probability proportional to the number of particles on each side.

In addition to $X_{t}$ satisfying dynamical equivariance, it is direct to see that $p\left(\sigma \cdot y_{t}|\sigma \cdot x_{t}\right)=p\left(y_{t}|x_{t}\right)$ also holds. This makes $\left(X_{t},Y_{t}\right)$ satisfy Eqs. 19A and 19B. Thus, by defining $V_{t}=\sum_{k=1}^{n}Y_{t}^{k}$, Theorem 2 shows that $\left(Z_{t},V_{t}\right)$ also constitute an HMM, so Corollary 3 guarantees that one can use $V_{t}$ to track the evolution of $Z_{t}$ via the abstract belief $b_{t}^{*}=p\left(z_{t}|{\overset{\leftarrow }{v}}_{t}\right)$. Crucially, the abstract beliefs $b_{t}^{*}$ are (n+1)-dimensional and hence are more efficient to compute and update than the micro-level beliefs $b_{t}$ (see Fig. 6D). That said, note that Eq. 26 is not satisfied in this scenario. Numerical evaluations were used to confirm the validity of Eq. 24.

In addition to the dynamics of the number of particles (which is the coarsest informationally self-contained level of the system), the Ehrenfest model has an intricate hierarchy of intermediate informationally self-contained levels corresponding to the subgroup lattice of $S_{n}$. Some of these subgroups correspond to treating certain particles as indistinguishable (i.e., allowing permutations only within specific blocks), which produces progressively coarser descriptions of the system in a similar way to how renormalization groups merge microscopic degrees of freedom into larger effective units. Thanks to Corollary 3, all these levels can also be tracked via coarse-grained beliefs, which can be updated using coarse-grained measurements that sum the estimation of particles in the corresponding blocks.

### Case study 3: Epidemic modeling

This final case study shows how dynamical symmetries can yield exact Bayesian inference of macroscopic variables in epidemic modeling. For this, consider a classic SIS model (*51*) in which recovered individuals can be reinfected. We will see how having a heterogeneous mixing makes the tracking of the total infection count challenging, but symmetries can be used to make this task computationally tractable.

For this, consider an epidemic process evolving on a population of *n* individuals, whose state at time *t* is given by $x_{t}=\left(x_{t}^{1},\ldots ,x_{t}^{n}\right)\in {\left\{0,1\right\}}^{n}$, where

$$x_{t}^{i}=\left\{\begin{array}{c} 1\;\text{if the}\;i\text{th}\;\text{individual is infected},\text{and}\\ 0\;\text{otherwise} \end{array}\right.$$ (31)

Assume that individuals are organized within *m* communities (*52*) (see Fig. 7A). Let $c_{i}\in \left\{1,\ldots ,m\right\}$ denote the community label of node *i*, and let the community infection counts be $z_{t}^{\ell }=\sum_{i:c_{i}=\ell }x_{t}^{i}$. Then, the probability of a susceptible individual being infected is modeled as (*53*)

$$p\left(x_{t+1}^{i}=1|x_{t}^{i}=0,x_{t}^{-i}\right)=1-e^{-h_{c_{i}}\left(z_{t}\right)}$$ (32)

where $x_{t}^{-i}=\left(x_{t}^{1},\ldots ,x_{t}^{i-1},x_{t}^{i+1},\ldots ,x_{t}^{n}\right)$ is a shorthand notation, $z_{t}=\left(z_{t}^{1},\ldots ,z_{t}^{m}\right)$, and $h_{c_{i}}\left(z_{t}\right)$ is the corresponding hazard (i.e., infection risk per unit time). Because of the community structure, the total risk of contagion for individuals within the ℓth community is modeled as

$$h_{\ell }\left(z_{t}\right)=\sum_{r=1}^{m}\beta_{\ell ,r}z_{t}^{r}$$ (33)

with $\beta_{\ell ,r}\leq \beta_{\ell ,\ell }$ accounting for the per-contact transmission rate for intercommunity interactions. In addition, the probability of an individual recovering is given by

$$p\left(x_{t+1}^{i}=0|x_{t}^{i}=1,x_{t}^{-i}\right)=\gamma$$ (34)

**Fig. 7.**
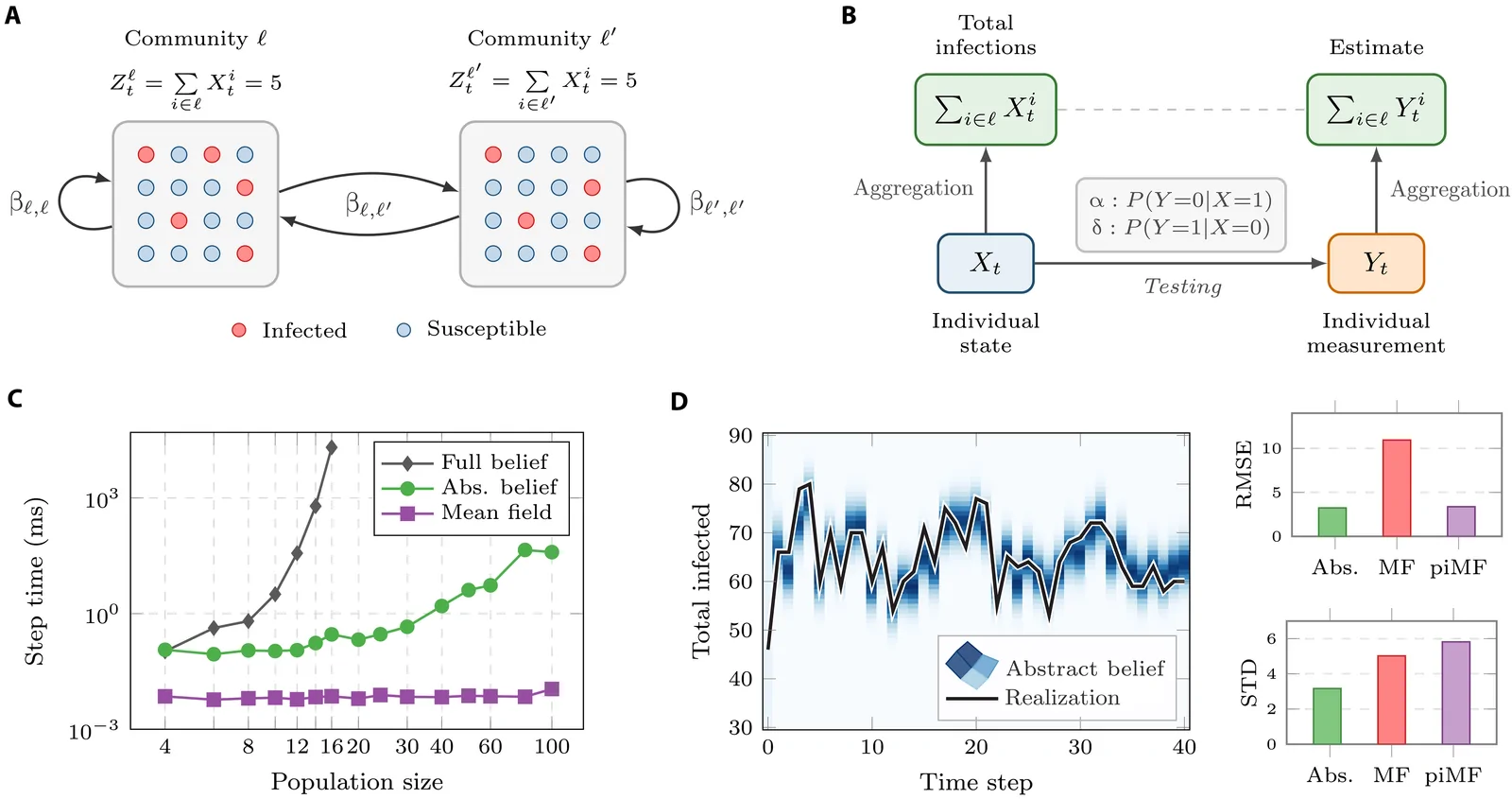
Symmetry yields exact Bayesian inference in epidemic modeling. (**A**) Microstates given by the status of individuals ($X_{t}^{i}$), and macrostates given by the number of infected subjects per community ($Z_{t}^{\ell }$). (**B**) Observations are made by measuring individuals via tests with false-negative rate α and false-positive rate δ. (**C**) Runtime per filtering step as a function of population size, comparing the updating of full beliefs $\left(p\left(x_{t}|{\overset{\leftarrow }{y}}_{t}\right)\right)$, abstract beliefs $\left(p\left(z_{t}|{\overset{\leftarrow }{v}}_{t}\right)\right)$, and a mean-field approximation for two equally sized communities. (**D**) Dynamics of the posterior of the total number of infections within two communities of sizes $n_{1}=120$ and $n_{2}=40$ using abstract beliefs, and comparison—in terms of root-mean-square error (RMSE) and standard deviation (SD)—against the posterior of a mean-field (MF) and a per-individual mean-field approximation (piMF).

Last, using Eqs. 32 and 34, the joint dynamics can be found to be given by

$$p\left(x_{t+1}|x_{t}\right)=\prod_{\left\{i:x_{t}^{i}=0\right\}}e^{\left(x_{t+1}^{i}-1\right)h_{c_{i}}\left(z_{t}\right)}{\left[1-e^{-h_{c_{i}}\left(z_{t}\right)}\right]}^{x_{t+1}^{i}}\times \prod_{\left\{i:x_{t}^{i}=1\right\}}{\left(1-\gamma \right)}^{x_{t+1}^{i}}\;\gamma^{1-x_{t+1}^{i}}$$ (35)

In line with epidemic monitoring scenarios (*54*), consider a setting where $x_{t}\in {\left\{0,1\right\}}^{n}$ can only be accessed via measurements $Y_{t}=\left(Y_{t}^{1},\ldots ,Y_{t}^{n}\right)$, where $Y_{t}^{k}\in \left\{0,1\right\}$ is a noisy estimate of $X_{t}^{k}$ (see Fig. 7B). Assume that individual tests are performed at each time point with false negative rate α and false positive rate δ, so that

$$p\left(y_{t}^{k}|x_{t}^{k}\right)={\left[{\left(1-\alpha \right)}^{x_{t}^{k}}\delta^{1-x_{t}^{k}}\right]}^{y_{t}^{k}}{\left[\alpha^{x_{t}^{k}}{\left(1-\delta \right)}^{1-x_{t}^{k}}\right]}^{1-y_{t}^{k}}$$ (36)

and $p\left(y_{t}|x_{t}\right)=\prod_{k=1}^{n}p\left(y_{t}^{k}|x_{t}^{k}\right)$. Using these measurements, one can build beliefs about $X_{t}$ of the form $b_{t}=p\left(x_{t}|{\overset{\leftarrow }{y}}_{t}\right)$. However, as with the Ehrenfest model, updating and storing such beliefs is computationally infeasible for reasonably large populations, with n>50 being beyond tractability.

Let us now show how symmetry can be used to yield exact and tractable Bayesian inference over macroscopic variables in this model. For this, let us denote the community sizes by $n_{1},\ldots ,n_{m}$, and consider the group $G=S_{n_{1}}\times \cdots \times S_{n_{m}}$ which acts by permuting coordinates within each community—i.e., by relabeling ${\left(g\cdot x\right)}^{\left(i\right)}≔x^{\sigma \left(i\right)}$, where σ permutes individuals’ indices while preserving community membership. A direct calculation shows that $p\left(g\cdot x_{t+1}|g\cdot x_{t}\right)=p\left(x_{t+1}|x_{t}\right)$, which implies that the system has equivariant dynamics. The recovery channel applies identically and independently to all infected nodes, while the infection channel for a susceptible node *i* depends on the current state *x* only through the community infection counts. A similar calculation shows that $p\left(g\cdot y_{t}|g\cdot x_{t}\right)=p\left(y_{t}|x_{t}\right)$, so that measurements are also equivariant. This, in turn, makes $\left(X_{t},Y_{t}\right)$ satisfy Eqs. 19A and 19B. The resulting orbits can be shown to correspond to $\phi_{G}\left(X_{t}\right)=Z_{t}=\left(Z_{t}^{1},\ldots ,Z_{t}^{m}\right)$ and $\psi_{G}\left(Y_{t}\right)=V_{t}=\left(V_{t}^{1},\ldots ,V_{t}^{m}\right)$, where

$$Z_{t}^{\ell }=\sum_{i:c_{i}=\ell }X_{t}^{i}\;\text{and}\;V_{t}^{\ell }=\sum_{i:c_{i}=\ell }Y_{t}^{i}$$ (37)

are the number of infected individuals per community. Thus, owing to Theorem 2, one can show that $\left(Z_{t},V_{t}\right)$ form an HMM over the reduced state space $\prod_{\ell =1}^{m}\left\{0,1,\ldots ,n_{\ell }\right\}$. Expressions for the dynamics and emission probabilities of this simplified HMM are derived in Methods. Defining the abstract belief $b_{t}^{*}=p\left(z_{t}|{\overset{\leftarrow }{v}}_{t}\right)$ allows one to perform inference over $Z_{t}$ without representing the $2^{n}$-dimensional belief $p\left(x_{t}|{\overset{\leftarrow }{y}}_{t}\right)$, replacing it with a belief of dimension $\prod_{\ell =1}^{m}\left(n_{\ell }+1\right)$.

To illustrate the benefits of symmetries in this scenario, we performed two experiments. First, the runtime per belief update on a standard laptop for populations with m=2 communities and increasing size *n* was calculated for three methods: full belief updating $\left(p\left(x_{t}|{\overset{\leftarrow }{y}}_{t}\right)\right)$, abstract belief updating $\left(p\left(z_{t}|{\overset{\leftarrow }{v}}_{t}\right)\right)$, and a mean-field estimation of the total number of infections (for details, see section S10). Results show that abstract beliefs have a much more gradual increase in runtime than full belief updating, being tractable for models of hundreds of individuals (see Fig. 7C). Second, the performance of abstract beliefs was tested for a heterogeneous population of *n* = 160 individuals grouped in two communities of sizes $n_{1}=120$ and $n_{2}=40$ (see section S10). Under these conditions, abstract belief updating was found to have a substantially better performance than a mean-field approximation (see Fig. 7D). The reason for the poor performance of a mean-field approximation is that, owing to the underlying heterogeneity, the dynamics of the total number of infections are not Markovian (see section S10). A better performance can be achieved by calculating separate mean-field approximations per individual. This approach achieves an average error comparable to abstract beliefs, but—because of neglecting interdependencies—provides a much broader posterior distribution.

Together, these results reveal how dynamical symmetries yield informationally self-contained macroscopic variables whose exact Bayesian beliefs can be updated efficiently. This enables tractable inference in partially observed systems, where micro-level Bayesian belief updating would be computationally infeasible.

## DISCUSSION

This paper combines ideas from information theory, group theory, and statistical mechanics to propose symmetry as a sufficient mechanism leading to hierarchical emergence and abstraction. Symmetries that commute with the dynamics give rise to simpler informationally self-contained levels of a process, revealing a whole range of emergent levels arranged in accordance with the subgroup lattice. Moreover, the hierarchy of emergent self-contained levels is reflected by Bayesian beliefs that can be hierarchically updated at various levels of resolution. Thus, symmetry not only structures the hierarchy of informationally self-contained levels of the process, but also organizes our inferences about them. Overall, this provides a theoretical framework to explain how dynamical processes give rise to hierarchical self-contained descriptions and effective theories, and how it is possible for observers to track macroscopic properties using noisy measurements via abstract belief updating.

### Symmetries, broken or factored

The results presented here shed light on the origins of hierarchical emergence, contributing to a better understanding of not only when it happens but also how it does so. These findings complement ongoing efforts to understand other flavors of emergence in dynamical processes focused on enhanced controllability (*55*, *56*) or whole-part relationships (*57*–*59*).

A prominent tradition holds that emergent phenomena (e.g., phase transitions, chirality, pattern formation, and the arrow of time) arise from broken symmetry (*5*, *60*, *61*). This is not in tension with the present account, but is complementary to it: Broken symmetry typically concerns symmetries of distributions over states, whereas the symmetries considered here are equivariances of the dynamics. In addition, the former approach focuses on symmetries related to statistics over trajectories, which can diverge from ensemble statistics when ergodicity is broken (*62*). Thus, the two approaches focus on different questions, leading to different criteria for what a “good coarse-graining” is. From a broken-symmetry perspective, coarse-grainings are chosen to distinguish phases; the approach proposed here instead looks for coarse-grainings that are informationally self-contained, being a statement about dynamics and not about phases.

Relatedly, renormalization group theory provides a complementary account of emergence by tracking how Hamiltonians change under repeated blocking and rescaling, explaining universality through fixed points and relevant or irrelevant perturbations (*63*). Symmetry plays a distinctive role in renormalization and effective field theory: It determines the compatible operators and provides constraints to the emergent laws. In contrast, symmetries in this work refer to dynamics and directly determine the form of the coarse-graining mappings. Thus, both approaches use symmetries to generate hierarchies but attain this via different procedures—a technical comparison of these approaches is provided in section S11. Future work may combine these frameworks, considering approaches that blend renormalization with principles of statistical inference (*64*), information theory (*65*, *66*), and variational methods (*67*).

The practical applicability of emergence as a way to build effective descriptions of systems of interest is supported by two empirical claims: the manifold hypothesis (*68*), which states that real-world high-dimensional data tend to concentrate near a low-dimensional submanifold, and the low-rank hypothesis (*69*), which states that interaction matrices of real complex systems tend to have a few dominant modes carrying most of the structure. However, a key challenge in making emergence useful for learning scenarios is designing efficient methods to identify emerging coarse-grained levels. In addition to renormalization-like heuristic methods (section S11), linear methods (*16*), or differentiable estimators (*70*), the results presented here suggest that symmetries could provide yet another route to do this. Future work may build on these results by designing statistical and machine learning methods to identify hierarchical emergence from data, extending the presented analysis of gene regulatory networks to more challenging settings. Key challenges associated with this include how to deal with finite samples and observational noise, and how to address settings with approximate symmetries (*71*, *72*).

### Beliefs that mirror the world

The correspondence between the hierarchical structure of systems of interest and beliefs of observers offers a principled explanation for the core tenet of geometric deep learning (*73*, *74*): that successful architectures should recapitulate the symmetries of the data they process (e.g., translation-equivariant layers in vision models). Such approaches presuppose that the data carry exploitable symmetries. The results presented here trace this to symmetries of the processes generating the data and show that these do not come alone but are arranged in a lattice—suggesting that deep learning architectures could recapitulate not merely a symmetry group but the whole hierarchy it induces.

The ideas investigated here are also related to old cybernetics ideas about how knowledge should resemble structural properties of its target (*75*, *76*), but arrive at these conclusions using modern tools of information theory and Bayesian filtering. Recent work also revisits cybernetics from a similar perspective but using category theory (*77*, *78*), exploring the relationship between external processes and epistemic beliefs in terms of functors and adjunctions. Future work may explore the potential of combining the presented framework with such techniques.

Overall, the presented results contribute toward a principled formalization of the notion of “abstraction,” which is a fundamental open problem in cognitive science (*79*–*81*) and artificial intelligence (*82*–*84*). There have been important advances formalizing abstractions in the context of reinforcement learning (*85*, *86*), including approaches leveraging symmetries (*87*), but most efforts have focused on scenarios where the environment is fully observable. While partially observable scenarios are formally solvable by turning them into fully observable problems by computing Bayesian beliefs (*88*), this approach does not work in practice because beliefs often are too high-dimensional. The approach presented here can be used to coarse-grain Bayesian beliefs in a principled manner, which could be used to address problems that are intractable to current methods. Furthermore, there is potential for exploring these ideas leveraging deep learning architectures focused on equivariance (*89*, *90*).

### Related work

The presented framework builds on a web of related but partially disconnected previous work, which is discussed next. Information closure was first introduced to quantify the autonomy of an organism with respect to its environment (*24*), and was later extended to probe the degree to which a macroscopic level depends on its underlying microscale (*25*). Previous work has used information closure to study emergence (*16*), but has not considered the role of symmetries. On the other hand, the reduction of Markov processes via lumpable partitions and other aggregation techniques is a well-investigated area with a rich literature (*91*–*95*). While the connection between lumpability and symmetry is known (*96*, *97*), the relationship between this and emergence has not been thoroughly explored. Lumpable HMMs have been investigated in (*98*), but their definition is more restricted than the one presented here, limiting their scope of applicability. Crucially, this work argues that lumpability corresponds to weak symmetries that are not adequate for characterizing hierarchical emergence in general, because they do not provide a structured hierarchy of partitions or bidirectional self-containment (see Methods for a technical discussion). The proposed distinction between weak, local, and global symmetries of Markov processes builds upon similar classifications of symmetries of graphs, as presented in (*31*), section 7.

Our approach to hierarchical belief updating is different from standard hierarchical Bayesian modeling (*99*), which focuses on hierarchies in parameter space and generally does not allow updating coarse descriptions without involving the fine-grained layers. In addition, the efficiency of abstract beliefs as proposed here differs from the approach used in variational inference (*100*), which simplifies the class of models considered at the cost of introducing approximation errors. In contrast, the method presented here achieves exact inference by carefully restricting the target of prediction.

Our approach is related to the work on collective graphical models (*101*, *102*), where inference is performed over population count statistics rather than trajectories of (independent) individuals. The framework presented here extends this to settings with interacting individuals and characterizes, via information closure, when exact aggregate filtering is available. Another related body of work is lifted inference, which uses first-order logic to build probabilistic graphical models with exchangeable variables that allow for inferences over equivalence classes rather than individuals (*103*–*106*). This literature has focused mainly on static models, but extensions to dynamic settings exist (*107*–*109*). While this literature focuses on symmetries of the target of prediction, the approach presented here also factors out symmetries in the measurements used to build beliefs—making the challenges faced by both approaches different. Also, while the lifted inference literature focuses on studying symmetries in the joint distribution, our approach to inference focuses on symmetries in the transition probabilities (Eqs. 19A and 19B). Given their complementarity, exploring ways to combine the strengths of lifted inference with the ideas presented here is an important direction for future work.

## METHODS

### Gene regulatory modeling

The studied Boolean model of the l-arabinose operon follows the one reported in (*48*). The model considers three external inputs: extracellular arabinose encoded by two binary indicators $\left(a_{e}^{h},a_{e}^{m}\right)$ with low=(0,0), medium=(0,1), and high=(1,1), unbound AraC (the regulatory protein controlling the l-arabinose operon) encoded by $a^{-}\in \left\{0,1\right\}$, and extracellular glucose encoded by $g_{e}\in \left\{0,1\right\}$. Our analyses focus on the case where the unbound AraC regulatory form is available ($a^{-}=1$), so that intracellular arabinose can generate the *A*^+^ regulatory signal, and the DNA looping mechanism *L* can respond to *A*^+^.

The synchronous Boolean update map $F:{\left\{0,1\right\}}^{9}\to {\left\{0,1\right\}}^{9}$ is as follows

$$\begin{array}{c} A_{t+1}=a_{e}^{h}T_{t},\\ A_{t+1}^{m}=a_{e}^{m}T_{t}\vee a_{e}^{h},\\ A_{t+1}^{+}=\left(A_{t}^{m}\vee A_{t}\right)a^{-},\\ C_{t+1}=1-g_{e},\\ E_{t+1}={\mathrm{MS}}_{t},\\ L_{t+1}=a^{-}\left(1-A_{t}^{+}\right),\\ {\mathrm{MS}}_{t+1}=A_{t}^{+}\left(1-L_{t}\right)C_{t},\\ {\mathrm{MT}}_{t+1}=A_{t}^{+}C_{t},\\ T_{t+1}={\mathrm{MT}}_{t} \end{array}$$

Above, all variables are binary, juxtaposition denotes Boolean AND, and ∨ denotes Boolean OR. This deterministic map was converted into a stochastic transition kernel by adding independent bit-flip noise after the synchronous update

$$M_{\mathrm{yx}}=p\left(X_{t+1}=y|X_{t}=x\right)={\left(1-\epsilon \right)}^{9-d\left(y,\;F\left(x\right)\right)}\epsilon^{d\left(y,\;F\left(x\right)\right)}$$

where d(⋅,⋅) is Hamming distance. The reported symmetries hold for any value of noise ϵ∈(0,1/2).

A symmetry of the stochastic dynamics is a permutation of the 2^9^ = 512 states that commutes with the transition probability matrix of $X_{t}$, corresponding to

$$\mathrm{PM}=\mathrm{MP}\;\text{for}\;P\in P_{512}$$ (38)

Computationally, this condition was evaluated by treating the transition matrix of $X_{t}$ as an edge-colored directed graph, where each microstate $x\in {\left\{0,1\right\}}^{9}$ is a vertex, and the edge x→y is colored by the transition weight $M_{\mathrm{yx}}=p\left(X_{t+1}=y|X_{t}=x\right)$. A brute force approach would be to test Eq. 38 for each permutation; however, this is computationally infeasible as $|P_{512}|\approx 3\times 10^{1166}$. While there is no efficient algorithm to find all lumpable partitions (to the best of my knowledge), global symmetries can often be found due to their algebraic properties.

The approach taken here is as follows:

1) Build a partition of the state space made of blocks with states with identical incoming and outgoing color signatures. States in different blocks are thus distinguishable after one step.

2) Refine this partition iteratively by comparing, for every candidate block, the multiset of edge colors sent to and received from every other block. Once the refinement stabilizes, states in different blocks are distinguishable after some finite number of steps.

3) The remaining question is whether states inside each block are freely exchangeable. This can be certified by testing transpositions within each block: If every swap of two states in a block preserves the full color matrix, then the automorphism group contains the full symmetric group on that block.

For the present Boolean model, step 2 produced blocks for which every within-block transposition preserves *M*, and therefore, the resulting automorphism group is

$$\text{Aut}\left(M\right)≅\prod_{i}S_{|B_{i}|}$$

where $B_{i}$ are the refined blocks with more than one element. Note that while this method succeeds in this model, it does not provide a universal way to find all global symmetries of transition matrices in general.

### Details of coarse-grained epidemic dynamics

Let us derive the dynamics of $Z_{t}$ by calculating $p\left(z_{t+1}|z_{t}\right)$. Conditioned on $Z_{t}=z_{t}$, the number of recoveries and new infections in the ℓth community at time point *t* distribute according to

$$R_{t}^{\ell }|\left(Z_{t}=z_{t}\right)∼\text{Binomial}\left(z_{t}^{\ell },\gamma \right)\;\text{and}$$ (39)

$$I_{t}^{\ell }|\left(Z_{t}=z_{t}\right)∼\text{Binomial}\left[n_{\ell }-z_{t}^{\ell },1-e^{-h_{\ell }\left(z_{t}\right)}\right]$$ (40)

so that $Z_{t+1}^{\ell }=Z_{t}^{\ell }-R_{t}^{\ell }+I_{t}^{\ell }$. Using this, one can calculate the transition probabilities for the dynamics of $Z_{t}$ as

$$\begin{array}{c} p\left(z_{t+1}|z_{t}\right)=\prod_{\ell =1}^{m}\sum_{r_{\ell }=r_{t}^{\ell ,\text{min}}}^{r_{t}^{\ell ,\text{max}}}\left(\begin{array}{c} z_{t}^{\ell }\\ r_{\ell } \end{array}\right)\gamma^{r_{\ell }}{\left(1-\gamma \right)}^{z_{t}^{\ell }-r_{\ell }}\times \\ \left(\begin{array}{c} n_{\ell }-z_{t}^{\ell }\\ \Delta_{t}^{\ell }+r_{\ell } \end{array}\right){\left[1-e^{-h_{\ell }\left(z_{t}\right)}\right]}^{\Delta_{t}^{\ell }+r_{\ell }}e^{-\left(n_{\ell }-z_{t+1}^{\ell }-r_{\ell }\right)h_{\ell }\left(z_{t}\right)} \end{array}$$ (41)

where $\Delta_{t}^{\ell }=z_{t+1}^{\ell }-z_{t}^{\ell }$, and summation limits (ensuring valid binomial arguments) are given by

$$r_{t}^{\ell ,\text{min}}=\text{max}\left(0,z_{t}^{\ell }-z_{t+1}^{\ell }\right),\;r_{t}^{\ell ,\text{max}}=\text{min}\left(z_{t}^{\ell },n_{\ell }-z_{t+1}^{\ell }\right)$$ (42)

Let us calculate the probability $p\left(v_{t}|z_{t}\right)$. Because communities partition individuals and test outcomes are conditionally independent given $X_{t}$, the joint emission distribution factorizes across communities so that $p\left(v_{t}|z_{t}\right)=\prod_{\ell =1}^{m}p\left(v_{t}^{\ell }|z_{t}^{\ell }\right)$ holds. Now, conditioned on $Z_{t}^{\ell }=z^{\ell }$, there are $z^{\ell }$ infected and $n_{\ell }-z^{\ell }$ uninfected individuals in that community. Let $A_{\ell }$ be the number of infected individuals that test positive and $B_{\ell }$ be the number of uninfected individuals that (falsely) test positive. Then

$$A_{\ell }|\left(Z_{t}^{\ell }=z^{\ell }\right)∼\text{Binomial}\left(z^{\ell },1-\alpha \right),B_{\ell }|\left(Z_{t}^{\ell }=z^{\ell }\right)∼\text{Binomial}\left(n_{\ell }-z^{\ell },\delta \right)$$ (43)

and $A_{\ell }$ and $B_{\ell }$ are independent conditional on $Z_{t}^{\ell }=z^{\ell }$. Moreover, $V_{t}^{\ell }=A_{\ell }+B_{\ell }$, and hence, the emission probability is the convolution of two binomials

$$p\left(v_{t}^{\ell }|z_{t}^{\ell }\right)=\sum_{a=a_{\text{min}}}^{a_{\text{max}}}\left(\begin{array}{c} z_{t}^{\ell }\\ a \end{array}\right){\left(1-\alpha \right)}^{a}\alpha^{z_{t}^{\ell }-a}\times \left(\begin{array}{c} n_{\ell }-z_{t}^{\ell }\\ v_{t}^{\ell }-a \end{array}\right)\delta^{v_{t}^{\ell }-a}{\left(1-\delta \right)}^{\left(n_{\ell }-z_{t}^{\ell }\right)-\left(v_{t}^{\ell }-a\right)}$$ (44)

where the summation limits ensure valid binomial arguments

$$a_{\text{min}}=\text{max}\left(0,v_{t}^{\ell }-\left(n_{\ell }-z_{t}^{\ell }\right)\right),a_{\text{max}}=\text{min}\left(z_{t}^{\ell },v_{t}^{\ell }\right)$$ (45)

### Weak symmetries in HMMs

The notion of weak symmetry can be extended from Markov processes to HMMs as follows. Consider two coarse-grainings ϕ:X→Z and ψ:Y→V, and let

$$Z_{t}=\phi \left(X_{t}\right)\;\text{and}\;V_{t}=\psi \left(Y_{t}\right)$$ (46)

Then, define the coarse-grained transition and emission probabilities as

$$\mu_{\phi }\left(z_{t+1}|x_{t}\right)≔\sum_{x_{t+1}\in \phi^{-1}\left(z_{t+1}\right)}p\left(x_{t+1}|x_{t}\right)$$ (47)

$$\nu_{\psi }\left(v_{t}|x_{t}\right)≔\sum_{y_{t}\in \psi^{-1}\left(v_{t}\right)}p\left(y_{t}|x_{t}\right)$$ (48)

We say that $\left(X_{t},Y_{t}\right)$ has a weak symmetry with respect to (ϕ,ψ), or equivalently that it is lumpable, if $\phi \left(x_{t}\right)=\phi \left(x_{t}^{\prime }\right)$ implies

$$\mu_{\phi }\left(z_{t+1}|x_{t}\right)=\mu_{\phi }\left(z_{t+1}|x_{t}^{\prime }\right)\forall z_{t+1}\in Z$$ (49A)

$$\nu_{\psi }\left(v_{t}|x_{t}\right)=\nu_{\psi }\left(v_{t}|x_{t}^{\prime }\right)\forall v_{t}\in V$$ (49B)

The first condition is the usual lumpability condition for the latent dynamics. The second requires the probabilities of the coarse-grained observations to be constant within every latent lump.

This condition has the following property.

Proposition 2. *If* $\left(X_{t},Y_{t}\right)$ *has a weak symmetry with respect to* (ϕ,ψ)*, then* $\left(Z_{t},V_{t}\right)$ *with* $Z_{t}=\phi \left(X_{t}\right)$ *and* $V_{t}=\psi \left(Y_{t}\right)$ *is an HMM.*

*Proof.* Suppose that Eqs. 49A and 49B hold. Then, a direct calculation shows that

$$p\left({\vec{z}}_{0}^{\;L+1},{\vec{v}}_{0}^{\;L}\right)=p\left(z_{0}\right)\prod_{\tau =0}^{L-1}\mu_{\phi }\left(z_{\tau +1}|z_{\tau }\right)\nu_{\psi }\left(v_{\tau }|z_{\tau }\right)$$ (50)

Hence, $\left(Z_{t},V_{t}\right)$ is an HMM. □

Every equivariant HMM induces such a weak symmetry by taking $\phi =\phi_{G}$ and $\psi =\psi_{G}$: summing Eqs. 19A and 19B over the corresponding orbits gives Eqs. 49A and 49B. The converse, however, does not hold. A weak symmetry only equates the total probability assigned to each coarse-grained cell. The probabilities of individual microscopic transitions and observations within that cell may differ and need not be related by any consistent relabeling.

Consequently, weak symmetry guarantees one exact quotient HMM and hence exact Bayesian filtering of $Z_{t}$ from $V_{t}$. It does not, in general, provide the additional structure supplied by equivariance: (i) there need not be a group whose subgroups generate a hierarchy of nested HMMs (Theorem 2 and Corollary 2), (ii) the fine-grained future trajectory trees need not be isomorphic (Proposition 1), and (iii) the same partition need not be lumpable for the reverse-time dynamics (see Corollary 1). Thus, weakly symmetric HMMs support self-contained inference at a specified coarse-grained level, but not the hierarchy or bidirectional guarantees associated with equivariant HMMs, as discussed next.

To study the bidirectional properties of HMMs more concretely, consider how one could estimate a Markov process $X_{t}$ based on future measurements, ${\vec{Y}}_{t}^{\;F-t+1}=\left(Y_{t},\ldots ,Y_{F}\right)$, using retrodictive Bayesian beliefs $D_{t}\;:=\;E\left\{1_{X_{t}}|{\vec{Y}}_{t}^{\;F-t+1}\right\}$, which when conditioned on a realization become $d_{t}\in {\mathbb{R}}^{|X|}$ with $d_{t}\left(x_{t}\right)=p\left(x_{t}|{\vec{y}}_{t}^{\;F-t+1}\right)$. Retrodictive beliefs can be updated via reversed-time Bayesian filtering (*49*)

$$d_{t}=\text{Norm}\text{[diag}\left(L_{y_{t}}\right)T_{t}^{†}d_{t+1}]≔U_{x,y}^{†}\left(y_{t},d_{t+1}\right)$$ (51)

where $T_{t}^{†}\in {\mathbb{R}}^{|X|\times |X|}$ is a transition probability matrix with components ${\left[T_{t}^{†}\right]}_{x_{t},x_{t+1}}=p\left(x_{t}|x_{t+1}\right)$. In addition, one can also estimate $X_{t}$ using both past and future observations ${\vec{Y}}_{0}^{\;F+1}$ with 0≤t≤F using smoothed Bayesian beliefs $\Omega_{t}\;:=E\left\{1_{X_{t}}|{\vec{Y}}_{0}^{\;F+1}\right\}$ that become $\omega_{t}\in {\mathbb{R}}^{|X|}$ with $\omega_{t}\left(x_{t}\right)=p\left(x_{t}|{\vec{y}}_{0}^{\;F+1}\right)$ when conditioned. These beliefs can be calculated as

$$\omega_{t}\left(x_{t}\right)=\text{Norm}\left[b_{t}\left(x_{t}\right)a_{t}\left(x_{t}\right)\right]$$ (52)

where $b_{t}\left(x_{t}\right)=p\left(x_{t}|{\overset{\leftarrow }{y}}_{t}\right)$ are the standard Bayesian beliefs and $a_{t}\left(x_{t}\right)=p\left({\vec{y}}_{t+1}^{\;F-t}|x_{t}\right)$. Smoothed beliefs can be calculated by updating $b_{t}$ via $b_{0}\to b_{1}\to \ldots$ using Eq. 21, and backward updating $a_{t}$ via $a_{F}\to a_{F-1}\to \ldots$ using

$$a_{t}=T_{t+1}^{⊺}\text{diag}\left(L_{y_{t+1}}\right)a_{t+1}$$ (53)

where $T_{t+1}^{⊺}$ is the transpose of $T_{t+1}$.

Although Proposition 2 guarantees that retrodictive and smoothed beliefs can also be calculated directly within the coarse-grained HMM $\left(Z_{t},V_{t}\right)$, weak symmetry does not guarantee that these calculations commute with coarse-graining the corresponding microscopic beliefs. Lumpability of the forward transition kernel $T_{t}$ does not, in general, imply lumpability of the reverse-time kernel $T_{t}^{†}$, which also depends on the distribution of $X_{t}$.

Consequently, the backward message $a_{t}\left(x_{t}\right)$ may distinguish microscopic states belonging to the same cell of ϕ, so that the coarse-graining of $d_{t}$ or $\omega_{t}$ cannot generally be propagated using only coarse-grained quantities. In particular, the retrodictive and smoothed beliefs obtained directly from $\left(Z_{t},V_{t}\right)$ need not coincide with $P_{\phi }d_{t}$ and $P_{\phi }\omega_{t}$, respectively. This compatibility is recovered when the same partition is also lumpable for the reverse-time dynamics, together with the corresponding sufficiency condition for the coarse-grained measurements. Global equivariance under marginal distributions that are invariant under the group action provides a sufficient condition for this additional reverse-time symmetry, whereas weak symmetry alone does not.

## Supplementary Materials

### This PDF file includes:

Sections S1 to S11 References

## References

[1] D. H. Wolpert, The lack of a priori distinctions between learning algorithms. Neural Comput. 8, 1341–1390 (1996).

[2] S. P. Adam, S.-A. N. Alexandropoulos, P. M. Pardalos, M. N. Vrahatis, “No free lunch theorem: A review,” in *Approximation and Optimization: Algorithms, Complexity and Applications* (Springer, 2019), pp. 57–82.

[3] S. C. Gibb, R. F. Hendry, T. Lancaster, Eds. *The Routledge Handbook of Emergence* (Routledge, 2019).

[4] S. M. Carroll, A. Parola, “What emergence can possibly mean,” in *Dennett’s Real Patterns in Science and Nature*, T. Millhouse, S. Petersen, D. Ross, Eds. (The MIT Press, 2026), pp. 113–136.

[5] D. C. Krakauer, Symmetry–simplicity, broken symmetry–complexity. Interface Focus 13, 20220075 (2023). 10.1098/rsfs.2022.0075 PMC10102721 37065260

[6] F. E. Rosas, B. C. Geiger, A. I. Luppi, A. K. Seth, D. Polani, M. Gastpar, P. A. M. Mediano, Software in the natural world: A computational approach to hierarchical emergence. arXiv:2402.09090 [nlin.AO] (2024).

[7] D. C. Krakauer, M. Mitchell, J. W. Krakauer, Large language models and emergence: A complex systems perspective. Philos. Trans. A Math. Phys. Eng. Sci. 384, 20250014 (2026). 10.1098/rsta.2025.0014 42130423

[8] P. W. Anderson, More is different: Broken symmetry and the nature of the hierarchical structure of science. Science 177, 393–396 (1972).

[9] L. P. Kadanoff, Scaling laws for Ising models near Tc. Phys. Phys. Fiz. 2, 263 (1966).

[10] K. G. Wilson, Problems in physics with many scales of length. Sci. Am. 241, 158–179 (1979).

[11] R. V. Solé, *Phase Transitions* (Princeton Univ. Press, 2011), vol. 16.

[12] N. Dupuis, L. Canet, A. Eichhorn, W. Metzner, J. M. Pawlowski, M. Tissier, N. Wschebor, The nonperturbative functional renormalization group and its applications. Phys. Rep. 910, 1–114 (2021).

[13] A. K. Seth, Measuring autonomy and emergence via Granger causality. Artif. Life 16, 179–196 (2010). 10.1162/artl.2010.16.2.16204 20067405

[14] E. P. Hoel, L. Albantakis, G. Tononi, Quantifying causal emergence shows that macro can beat micro. Proc. Natl. Acad. Sci. U.S.A. 110, 19790–19795 (2013). 10.1073/pnas.1314922110 PMC3856819 24248356

[15] F. E. Rosas, P. A. M. Mediano, H. J. Jensen, A. K. Seth, A. B. Barrett, R. L. Carhart-Harris, D. Bor, Reconciling emergences: An information-theoretic approach to identify causal emergence in multivariate data. PLOS Comput. Biol. 16, e1008289 (2020). 10.1371/journal.pcbi.1008289 PMC7833221 33347467

[16] L. Barnett, A. K. Seth, Dynamical independence: Discovering emergent macroscopic processes in complex dynamical systems. Phys. Rev. E 108, 014304 (2023). 10.1103/PhysRevE.108.014304 37583178

[17] M. A. Bedau, P. Humphreys, Eds. *Emergence: Contemporary Readings in Philosophy and Science* (MIT Press, 2008).

[18] P. Clayton, P. Davies, Eds. *The Re-emergence of Emergence: The Emergentist Hypothesis from Science to Religion* (Oxford Univ. Press, 2006).

[19] H. J. Jensen, *Complexity Science: The Study of Emergence* (Cambridge Univ. Press, 2022).

[20] J. P. Crutchfield, The calculi of emergence: Computation, dynamics and induction. Phys. D Nonlinear Phenom. 75, 11–54 (1994).

[21] N. Israeli, N. Goldenfeld, Coarse-graining of cellular automata, emergence, and the predictability of complex systems. Phys. Rev. E 73, 026203 (2006). 10.1103/PhysRevE.73.026203 16605425

[22] D. Krakauer, N. Bertschinger, E. Olbrich, J. C. Flack, N. Ay, The information theory of individuality. Theory Biosci. 139, 209–223 (2020). 10.1007/s12064-020-00313-7 PMC7244620 32212028

[23] C. P. Kempes, D. C. Krakauer, The multiple paths to multiple life. J. Mol. Evol. 89, 415–426 (2021). 10.1007/s00239-021-10016-2 PMC8318961 34254169

[24] N. Bertschinger, E. Olbrich, N. Ay, J. Jost, “Information and closure in systems theory,” in *Explorations in the Complexity of Possible Life. Proceedings of the 7th German Workshop of Artificial Life* (IOS Press, 2006), pp. 9–21.

[25] A. Y. C. Chang, M. Biehl, Y. Yen, R. Kanai, Information closure theory of consciousness. Front. Psychol. 11, 1504 (2020). 10.3389/fpsyg.2020.01504 PMC7374725 32760320

[26] K. H. G. Vollbrecht, R. F. Werner, Entanglement measures under symmetry. Phys. Rev. A 64, 062307 (2001).

[27] A. Kolchinsky, D. H. Wolpert, Work, entropy production, and thermodynamics of information under protocol constraints. Phys. Rev. X 11, 041024 (2021).

[28] O. Reynolds, On the dynamical theory of incompressible viscous fluids and the determination of the criterion. Phil. Trans. R. Soc. A 186, 123–164 (1895).

[29] M. J. Field, Equivariant dynamical systems. Trans. Am. Math. Soc. 259, 185–205 (1980).

[30] M. Golubitsky, I. Stewart, D. G. Schaeffer, *Singularities and Groups in Bifurcation Theory: Volume II*, volume 69 (Springer Science & Business Media, 2012).

[31] H. A. Makse, P. Boldi, F. Sorrentino, I. Stewart, Symmetries of living systems: Symmetry fibrations and synchronization in biological networks. arXiv:2502.18713 [q-bio.MN] (2025).

[32] J. G. Kemeny, J. L. Snell, *Finite Markov Chains* (van Nostrand Princeton, 1969).

[33] S.-I. Amari, Learning patterns and pattern sequences by self-organizing nets of threshold elements. IEEE Trans. Comput. 11, 1197–1206 (1972).

[34] J. J. Hopfield, Neural networks and physical systems with emergent collective computational abilities. Proc. Natl. Acad. Sci. U.S.A. 79, 2554–2558 (1982). 10.1073/pnas.79.8.2554 PMC346238 6953413

[35] G. E. Hinton, T. J. Sejnowski, “Learning and relearning in Boltzmann machines,” in *Parallel Distributed Processing: Explorations in the Microstructure of Cognition*, *Volume 1: Foundations*, D. E. Rumelhart, J. L. McClelland, the PDP Research Group, Eds. (MIT Press, 1986), chap. 7, pp. 282–317.

[36] D. Krotov, A new frontier for Hopfield networks. Nat. Rev. Phys. 5, 366–367 (2023).

[37] M. Aguilera, P. A. Morales, F. E. Rosas, H. Shimazaki, Explosive neural networks via higher-order interactions in curved statistical manifolds. Nat. Commun. 16, 6511 (2025). 10.1038/s41467-025-61475-w PMC12290047 40707463

[38] D. J. Amit, *Modeling Brain Function: The World of Attractor Neural Networks* (Cambridge Univ. Press, 1989).

[39] W. Gerstner, W. M. Kistler, R. Naud, L. Paninski, *Neuronal Dynamics: From Single Neurons to Networks and Models of Cognition* (Cambridge Univ. Press, 2014).

[40] D. O. Hebb, *The Organization of Behavior* (Wiley & Sons, 1949). 10.1016/s0361-9230(99)00182-3 10643472

[41] A. Barra, M. Beccaria, A. Fachechi, A new mechanical approach to handle generalized Hopfield neural networks. Neural Netw. 106, 205–222 (2018). 10.1016/j.neunet.2018.07.010 30081347

[42] E. Agliari, A. Alessandrelli, A. Barra, F. Ricci-Tersenghi, Parallel learning by multitasking neural networks. J. Stat. Mech. Theory Exp. 2023, 113401 (2023).

[43] S. A. Kauffman, Metabolic stability and epigenesis in randomly constructed genetic nets. J. Theor. Biol. 22, 437–467 (1969). 10.1016/0022-5193(69)90015-0 5803332

[44] S. Bornholdt, Boolean network models of cellular regulation: Prospects and limitations. J. R. Soc. Interface 5, S85 (2008). 10.1098/rsif.2008.0132.focus PMC2386560 18508746

[45] A. Saadatpour, R. Albert, Boolean modeling of biological regulatory networks: A methodology tutorial. Methods 62, 3–12 (2013). 10.1016/j.ymeth.2012.10.012 23142247

[46] W. Abou-Jaoudé, P. Traynard, P. T. Monteiro, J. Saez-Rodriguez, T. Helikar, D. Thieffry, C. Chaouiya, Logical modeling and dynamical analysis of cellular networks. Front. Genet. 7, 94 (2016). 10.3389/fgene.2016.00094 PMC4885885 27303434

[47] I. Shmulevich, E. R. Dougherty, W. Zhang, Gene perturbation and intervention in probabilistic Boolean networks. Bioinformatics 18, 1319–1331 (2002). 10.1093/bioinformatics/18.10.1319 12376376

[48] A. Jenkins, M. Macauley, Bistability and asynchrony in a Boolean model of the L-arabinose operon in *Escherichia coli*. Bull. Math. Biol. 79, 1778–1795 (2017). 10.1007/s11538-017-0306-1 28639170

[49] S. Särkkä, L. Svensson, *Bayesian Filtering and Smoothing* (Cambridge Univ. Press, 2023), vol. 17.

[50] P. Ehrenfest, T. Ehrenfest-Afanassjewa, Über zwei bekannte Einwände gegen das Boltzmannsche H-Theorem. Phys. Z. 8, 311–314 (1907).

[51] H. W. Hethcote, The mathematics of infectious diseases. Siam Rev. 42, 599–653 (2000).

[52] R. Pastor-Satorras, C. Castellano, P. Van Mieghem, A. Vespignani, Epidemic processes in complex networks. Rev. Mod. Phys. 87, 925–979 (2015).

[53] L. J. S. Allen, “An introduction to stochastic epidemic models,” in *Mathematical Epidemiology* (Springer, 2008), pp. 81–130.

[54] R. Brookmeyer, D. F. Stroup, *Monitoring the Health of Populations: Statistical Principles and Methods for Public Health Surveillance* (Oxford Univ. Press, 2004).

[55] E. Hoel, Quantifying emergent complexity. Patterns 7, 101472 (2026). 10.1016/j.patter.2025.101472 PMC12827727 41583978

[56] A. Jansma, E. Hoel, Engineering emergence. arXiv:2510.02649 [cs.IT] (2025).

[57] T. F. Varley, Flickering emergences: The question of locality in information-theoretic approaches to emergence. Entropy 25, 54 (2023). 10.3390/e25010054 PMC9858457 36673195

[58] H. M. Tolle, A. I. Luppi, A. K. Seth, P. A. M. Mediano, Evolving reservoir computers reveal bidirectional coupling between predictive power and emergent dynamics. Patterns 7, 101457 (2026). 10.1016/j.patter.2025.101457 PMC13100675 42028408

[59] F. Pigozzi, A. Goldstein, M. Levin, Associative conditioning in gene regulatory network models increases integrative causal emergence. Commun. Biol. 8, 1027 (2025). 10.1038/s42003-025-08411-2 PMC12241368 40634498

[60] P. W. Anderson, “Some general thoughts about broken symmetry,” in *A Career in Theoretical Physics* (World Scientific, 1994), pp. 419–429.

[61] M. Fruchart, R. Hanai, P. B. Littlewood, V. Vitelli, Non-reciprocal phase transitions. Nature 592, 363–369 (2021). 10.1038/s41586-021-03375-9 33854249

[62] R. G. Palmer, Broken ergodicity. Adv. Phys. 31, 669–735 (1982).

[63] R. W. Batterman, *The Devil in the Details: Asymptotic Reasoning in Explanation, Reduction, and Emergence* (Oxford Univ. Press, 2001).

[64] D. S. Berman, M. S. Klinger, A. G. Stapleton, Bayesian renormalization. Mach. Learn. Sci. Technol. 4, 045011 (2023).

[65] M. Koch-Janusz, Z. Ringel, Mutual information, neural networks and the renormalization group. Nat. Phys. 14, 578–582 (2018).

[66] P. M. Lenggenhager, D. E. Gökmen, Z. Ringel, S. D. Huber, M. Koch-Janusz, Optimal renormalization group transformation from information theory. Phys. Rev. X 10, 011037 (2020).

[67] K. Friston, C. Heins, T. Verbelen, L. Da Costa, T. Salvatori, D. Markovic, A. Tschantz, M. Koudahl, C. Buckley, T. Parr, From pixels to planning: Scale-free active inference. Front. Netw. Physiol. 5, 1521963 (2025). 10.3389/fnetp.2025.1521963 PMC12217590 40607319

[68] C. Fefferman, S. Mitter, H. Narayanan, Testing the manifold hypothesis. J. Am. Math. Soc. 29, 983–1049 (2016).

[69] V. Thibeault, A. Allard, P. Desrosiers, The low-rank hypothesis of complex systems. Nat. Phys. 20, 294–302 (2024).

[70] D. McSharry, C. Kaplanis, F. E. Rosas, P. A. Mediano, Learning diverse causally emergent representations from time series data. Adv. Neural Inf. Proces. Syst. 37, 119547–119572 (2024).

[71] H. Charvin, N. C. Volpi, D. Polani, Towards information theory-based discovery of equivariances. arXiv:2310.16555 [cs.IT] (2023).

[72] F. E. Rosas, Adaptive state-action abstractions via rate-distortion. arXiv:2606.06123 [cs.LG] (2026).

[73] M. M. Bronstein, J. Bruna, T. Cohen, P. Veličković, Geometric deep learning: Grids, groups, graphs, geodesics, and gauges. arXiv:2104.13478 [cs.LG] (2021).

[74] L.-H. Lim, B. J. Nelson, What is an equivariant neural network? Not. Am. Math. Soc. 70, 619–625 (2023).

[75] R. C. Conant, W. R. Ashby, Every good regulator of a system must be a model of that system. Int. J. Syst. Sci. 1, 89–97 (1970).

[76] W. R. Ashby, J. Goldstein, Variety, constraint, and the law of requisite variety. Emergence: Complexity and Organization 13, 190 (2011).

[77] N. Virgo, M. Biehl, M. Baltieri, M. Capucci, A “good regulator theorem” for embodied agents. arXiv:2508.06326 [cs.AI] (2025).

[78] M. Baltieri, M. Biehl, M. Capucci, N. Virgo, A Bayesian interpretation of the internal model principle. arXiv:2503.00511 [math.OC] (2025).

[79] M. K. Ho, D. Abel, T. L. Griffiths, M. L. Littman, The value of abstraction. Curr. Opin. Behav. Sci. 29, 111–116 (2019).

[80] M. K. Ho, D. Abel, C. G. Correa, M. L. Littman, J. D. Cohen, T. L. Griffiths, People construct simplified mental representations to plan. Nature 606, 129–136 (2022). 10.1038/s41586-022-04743-9 35589843

[81] B. De Martino, A. Cortese, Goals, usefulness and abstraction in value-based choice. Trends Cogn. Sci. 27, 65–80 (2023). 10.1016/j.tics.2022.11.001 36446707

[82] F. Chollet, On the measure of intelligence. arXiv:1911.01547 [cs.AI] (2019).

[83] A. Geiger, D. Ibeling, A. Zur, M. Chaudhary, S. Chauhan, J. Huang, A. Arora, Z. Wu, N. Goodman, C. Potts, T. Icard, Causal abstraction: A theoretical foundation for mechanistic interpretability. J. Mach. Learn. Res. 26, 1–64 (2025).

[84] G. Konidaris, On the necessity of abstraction. Curr. Opin. Behav. Sci. 29, 1–7 (2019). 10.1016/j.cobeha.2018.11.005 PMC6706087 31440528

[85] D. Abel, “A theory of abstraction in reinforcement learning,” thesis, Brown University (2020).

[86] C. Allen, “Structured abstractions for general-purpose decision making,” thesis, Brown University (2023).

[87] E. Van der Pol, D. Worrall, H. van Hoof, F. Oliehoek, M. Welling, MDP homomorphic networks: Group symmetries in reinforcement learning. Adv. Neural Inf. Proces. Syst. 33, 4199–4210 (2020).

[88] L. P. Kaelbling, M. L. Littman, A. R. Cassandra, Planning and acting in partially observable stochastic domains. Artif. Intell. 101, 99–134 (1998).

[89] A. Di Bernardo, A. Valente, F. Mastrogiuseppe, S. Ostojic, Shaping Slow Manifolds in Equivariant Recurrent Neural Networks. Neural Comput. 38, 1913–1981 (2026). 10.1162/NECO.a.1577 42771685

[90] T. A. Keller, Flow equivariant recurrent neural networks. arXiv:2507.14793 [cs.LG] (2025).

[91] P. Buchholz, Exact and ordinary lumpability in finite Markov chains. J. Appl. Prob. 31, 59–75 (1994).

[92] B. C. Geiger, T. Petrov, G. Kubin, H. Koeppl, Optimal Kullback–Leibler aggregation via information bottleneck. IEEE Trans. Automat. Contr. 60, 1010–1022 (2015).

[93] B. C. Geiger, C. Temmel, Lumpings of Markov chains, entropy rate preservation, and higher-order lumpability. J. Appl. Probab. 51, 1114–1132 (2014).

[94] M. Faccin, M. T. Schaub, J.-C. Delvenne, State aggregations in Markov chains and block models of networks. Phys. Rev. Lett. 127, 078301 (2021). 10.1103/PhysRevLett.127.078301 34459654

[95] A. C. Costa, T. Ahamed, D. Jordan, G. J. Stephens, Maximally predictive states: From partial observations to long timescales. Chaos 33, 023136 (2023). 10.1063/5.0129398 36859220

[96] P. L. Simon, M. Taylor, I. Z. Kiss, Exact epidemic models on graphs using graph-automorphism driven lumping. J. Math. Biol. 62, 479–508 (2011). 10.1007/s00285-010-0344-x PMC7079990 20425114

[97] S. Banisch, R. Lima, Markov chain aggregation for simple agent-based models on symmetric networks: The voter model. Adv. Complex Syst. 18, 1550011 (2015).

[98] L. B. White, R. Mahony, G. D. Brushe, Lumpable hidden Markov models—Model reduction and reduced complexity filtering. IEEE Trans. Automat. Contr. 45, 2297–2306 (2000).

[99] R. M. Shiffrin, M. D. Lee, W. Kim, E.-J. Wagenmakers, A survey of model evaluation approaches with a tutorial on hierarchical Bayesian methods. Cognit. Sci. 32, 1248–1284 (2008). 10.1080/03640210802414826 21585453

[100] D. M. Blei, A. Kucukelbir, J. D. McAuliffe, Variational inference: A review for statisticians. J. Am. Stat. Assoc. 112, 859–877 (2017).

[101] D. R. Sheldon, T. Dietterich, Collective graphical models. Adv. Neural Inf. Proces. Syst. 24, 9 (2011).

[102] D. Sheldon, T. Sun, A. Kumar, T. Dietterich, Approximate inference in collective graphical models, in *International Conference on Machine Learning*, (PMLR, 2013), pp. 1004–1012.

[103] D. Poole, “First-order probabilistic inference,” in IJCAI’03: *Proceedings of the 18th International Joint Conference on Artificial intelligence* (Morgan Kaufmann Publishers Inc., 2003), pp. 985–991.

[104] R. de Salvo Braz, E. Amir, D. Roth, “Lifted first-order probabilistic inference,” in *Proceedings of the Nineteenth International Joint Conference on Artificial Intelligence* (Morgan Kaufmann, 2005), pp. 1319–1325.

[105] B. Milch, L. S. Zettlemoyer, K. Kersting, M. Haimes, L. P. Kaelbling, “Lifted probabilistic inference with counting formulas,” in *AAAI’08: Proceedings of the 23rd National Conference on Artificial intelligence* (AAAI, 2008), pp. 1062–1068.

[106] M. Niepert, G. Van den Broeck, “Tractability through exchangeability: A new perspective on efficient probabilistic inference,” in *AAAI’14: Proceedings of the Twenty-Eighth AAAI Conference on Artificial Intelligence* (AAAI, 2014), pp. 2467–2475.

[107] S. Lüdtke, M. Schröder, F. Krüger, S. Bader, T. Kirste, State-space abstractions for probabilistic inference: A systematic review. J. Artif. Intell. Res. 63, 789–848 (2018).

[108] M. Schröder, S. Lüdtke, S. Bader, F. Krüger, T. Kirste, “LiMa: Sequential lifted marginal filtering on multiset state descriptions,” in *Joint German/Austrian Conference on Artificial Intelligence (Künstliche Intelligenz)* (Springer, 2017), pp. 222–235.

[109] M. Gehrke, T. Braun, R. Möller, “Lifted dynamic junction tree algorithm,” in *International Conference on Conceptual Structures* (Springer, 2018), pp. 55–69.

[110] I. Kanter, H. Sompolinsky, Associative recall of memory without errors. Phys. Rev. A Gen. Phys. 35, 380 (1987). 10.1103/physreva.35.380 9897963

