# Supplementary material for: Symmetry as a common origin of hierarchical emergence and abstraction
Source: Sci Adv. 2026 Nov 13;12(46):eaeh0773. doi: 10.1126/sciadv.aeh0773 (PMC13763957; doi:10.1126/sciadv.aeh0773)
Supplement: Supplementary file 1 [file sciadv.aeh0773_sm.pdf]

Science Advances
AAAS

Supplementary Materials for

Symmetry as a common origin of hierarchical emergence and abstraction

Fernando E. Rosas

Corresponding author: Fernando E. Rosas, f.rosas@sussex.ac.uk

Sci. Adv. **12**, eaeh0773 (2026)
DOI: 10.1126/sciadv.aeh0773

This PDF file includes:

Sections S1 to S11
References

Supplementary Material S1: Proof of Theorem 1

Proof. We need to prove that $Z_t = \phi_G(X_t)$ is informationally closed if and only if $\mathscr{R}\{K^L_{t+1}\}(\vec{\boldsymbol{g}}_t \cdot \vec{\boldsymbol{x}}_t) = \mathscr{R}\{K^L_{t+1}\}(\vec{\boldsymbol{x}}_t)$ for all $\vec{\boldsymbol{g}}_t$ and all $t \in \mathscr{Z}$ and $L \in \mathbb{N}$. If $Z_t = \phi_G(X_t)$ is given by the group orbits, then

$$p(\vec{\boldsymbol{z}}^L_{t+1}|\vec{\boldsymbol{x}}_t) = \sum_{\vec{\boldsymbol{x}}^L_{t+1} \in \phi^{-1}_{G^L}(\vec{\boldsymbol{z}}^L_{t+1})} p(\vec{\boldsymbol{x}}^L_{t+1}|\vec{\boldsymbol{x}}_t) \tag{S11}$$

$$\overset{(a)}{=} \left|\phi^{-1}_{G^L}(\vec{\boldsymbol{z}}^L_{t+1})\right| \mathscr{R}\left\{K^L_{t+1}\right\}(\vec{\boldsymbol{x}}_t), \tag{S12}$$

where (a) is a consequence of the orbit-stabiliser theorem. Thus, $p(\vec{\boldsymbol{z}}^L_{t+1}|\vec{\boldsymbol{x}}_t) \propto \mathscr{R}\left\{K^L_{t+1}\right\}(\vec{\boldsymbol{x}}_t)$, and hence $\mathscr{R}\left\{K^L_{t+1}\right\}(\vec{\boldsymbol{x}}_t)$ is invariant under component-wise action if and only if $p(\vec{\boldsymbol{z}}^L_{t+1}|\vec{\boldsymbol{x}}_t)$ is. The latter property holds if and only if Eq. 2 holds. Putting these equivalences together proves the desired result. □

Supplementary Material S2: Proof of Prop. 1

Proof. Denote as $\mathscr{T}^+_L(x)$ the depth-L forward trajectory tree rooted at x. Its vertices at depth ℓ are trajectories

$$(x_0, \dots, x_\ell), \qquad x_0 = x, \tag{S21}$$

and the edge obtained by appending $x_{\ell+1}$ has weight $p(x_{\ell+1}|x_\ell)$. Similarly, let $\mathscr{T}^-_L(x)$ denote the backward trajectory tree, whose vertices are trajectories

$$(x_{-\ell}, \dots, x_0), \qquad x_0 = x, \tag{S22}$$

and whose edge obtained by prepending $x_{-\ell-1}$ has weight $p(x_{-\ell}|x_{-\ell-1})$. An isomorphism of trajectory trees is a root-, depth-, adjacency-, and edge-weight-preserving bijection.

Let us first consider a global symmetry and suppose that $x' = g\cdot x$. Define

$$F^+_g(x_0, \dots, x_\ell) := (g\cdot x_0, \dots, g\cdot x_\ell). \tag{S23}$$

By Eq. 6, $p(g\cdot x_{j+1}|g\cdot x_j) = p(x_{j+1}|x_j)$, so F^+_g preserves every edge weight. Moreover, $F^+_{g^{-1}}$ is its inverse. Hence $\mathscr{T}^+_L(x) \cong \mathscr{T}^+_L(x')$. Applying the same component-wise map to backward trajectories gives

$$F^-_g(x_{-\ell}, \dots, x_0) := (g\cdot x_{-\ell}, \dots, g\cdot x_0). \tag{S24}$$

Again, Eq. 6 gives $p(g\cdot x_j \mid g\cdot x_{j-1}) = p(x_j \mid x_{j-1})$, and therefore $\mathscr{T}^-_L(x) \cong \mathscr{T}^-_L(x')$. Since this holds for every $L \in \mathbb{N}$, the complete past and future trajectory trees are isomorphic.

Now consider a local symmetry with associated partition $\mathscr{C}$, and write $u \sim v$ if $u, v \in \mathscr{X}$ are in the same cell. Let us prove by induction on L that for every $u, v \in \mathscr{X}$ such that $u \sim v$, there exists a tree isomorphism $F^{u,v}_L : \mathscr{T}^+_L(u) \to \mathscr{T}^+_L(v)$ that satisfies

$$F^{u,v}_L(u, x_1, \dots, x_L) = (v, y_1, \dots, y_L), \tag{S25}$$

with $x_k \sim y_k$ for every $k = 1, \dots, L$. For $L = 0$, both trees contain only their roots, and the map $u \mapsto v$ establishes the claim. Suppose now that the claim holds at depth L, and take $u, v \in \mathscr{X}$ such that $u \sim v$. For every child w of u, let B be the cell containing w and set $w' = \sigma^B_{u,v}(w)$. Then $w \sim w'$ and

$$p(w \mid u) = p(w' \mid v) \tag{S26}$$

because of Eq. 7. The induction hypothesis can be used to provide an isomorphism

$$F^{w,w'}_L : \mathscr{T}^+_L(w) \longrightarrow \mathscr{T}^+_L(w'). \tag{S27}$$

Define $F^{u,v}_{L+1}$ by mapping u to v, mapping each first-level child w to w', and applying $F^{w,w'}_L$ to the subtree rooted at w. The resulting map is bijective because each $\sigma^B_{u,v}$ and each $F^{w,w'}_L$ is bijective. It preserves the weights of the first-level edges by Eq. 7, and all subsequent edge weights by the induction hypothesis. It also preserves partition cells. Hence $\mathscr{T}^+_{L+1}(u) \cong \mathscr{T}^+_{L+1}(v)$.

Finally, consider a weak symmetry given by a coarse-graining $\phi(X) = Z$, associated with a lumpable partition. Lumpability ensures that

$$\mu_\phi(z_{t+1}|z_t) := \sum_{x_{t+1} \in \phi^{-1}(z_{t+1})} p(x_{t+1}|x_t) \tag{S28}$$

is well-defined, being independent of the representative $x_t \in \phi^{-1}(z_t)$. Thus, μ_ϕ is a well-defined transition kernel on the coarse-grained state space. In particular, for every coarse-grained trajectory $z_t, \dots, z_{t+L}$,

$$p(z_{t+1}, \dots, z_{t+L}|x_t) = \prod_{\ell=t}^{t+L-1} \mu_\phi(z_{\ell+1}|z_\ell), \tag{S29}$$

where $z_t = \phi(x_t)$. The right-hand side depends on x_t only through z_t. Hence, if $\phi(x_t) = \phi(x'_t)$, the rooted weighted trees of future coarse-grained trajectories generated from x_t and x'_t coincide, and are therefore isomorphic. □

Supplementary Material S3: Proof of Cor. 1

Proof. Suppose first that $Z_t = \phi(X_t)$ is induced by a weak symmetry, or equivalently, that its partition is lumpable. The quantity

$$\mu_\phi(z_{t+1}|z_t) := \sum_{x_{t+1} \in \phi^{-1}(z_{t+1})} p(x_{t+1}|x_t), \tag{S31}$$

is then independent of the representative $x_t \in \phi^{-1}(z_t)$, and defines a transition kernel on the coarse-grained state space $\mathscr{Z}$. By the Markov property and Eq. S31,

$$p\big(\vec{\boldsymbol{z}}^L_{t+1} \mid \vec{\boldsymbol{x}}_t\big) = p\big(\vec{\boldsymbol{z}}^L_{t+1} \mid x_t\big) = \prod_{\ell=0}^{L-1} \mu_\phi(z_{t+\ell+1}|z_{t+\ell}) \tag{S32}$$

for every microscopic past $\bar{\boldsymbol{x}}_t$ and every coarse-grained future $\vec{\boldsymbol{z}}^L_{t+1}$, where $z_t = \phi(x_t)$. The right-hand side depends on the microscopic past only through z_t, and therefore

$$p\big(\vec{\boldsymbol{z}}^L_{t+1} \mid \bar{\boldsymbol{x}}_t\big) = p\big(\vec{\boldsymbol{z}}^L_{t+1} \mid \bar{\boldsymbol{z}}_t\big). \tag{S33}$$

By Eq. 2, this implies that Z_t is informationally closed.

Conversely, suppose that Z_t is informationally closed and Markovian. Consider a pair $x_t, x'_t \in \mathrm{supp}(\mathscr{X}_t)$ such that $\phi(x_t) = \phi(x'_t) = z_t$. Applying informational closure with $L = 1$ gives

$$\sum_{x_{t+1}\in\phi^{-1}(z_{t+1})} p(x_{t+1}|x_t) = p(z_{t+1}|x_t) = p(z_{t+1}|z_t) \tag{S34}$$

$$= p(z_{t+1}|x'_t) = \sum_{x_{t+1}\in\phi^{-1}(z_{t+1})} p(x_{t+1}|x'_t). \tag{S35}$$

for every $z_{t+1} \in \mathscr{Z}$. Thus, the transition probability from a microscopic state to each cell of the partition depends only on the cell containing that state. If X_t has full support at some time t_0, the same equality holds for all $x, x' \in \mathscr{X}$, yielding the condition in Eq. 8, so ϕ is induced by a weak symmetry.

It remains to establish the reverse-time statement. Suppose that $p(x_{t+1}|x_t)$ is equivariant under G and that the process has G-invariant marginal distributions $p(x_t)$ and $p(x_{t+1})$. If $p(x_{t+1}) > 0$, then the reverse-time transition kernel is

$$p(x_t|x_{t+1}) = \frac{p(x_t)p(x_{t+1}|x_t)}{p(x_{t+1})}, \tag{S36}$$

which is itself G-equivariant. Its orbit partition is therefore lumpable for the reverse-time kernel, and the first part of the proof shows that the corresponding coarse-graining is informationally closed under time reversal. □

Supplementary Material S4: Proof of Cor. 2

Proof. The first part of the result follows directly from the fact that if X_t is equivariant with respect to G, it is also equivariant with respect to any subgroup H, and hence Cor. 1 also applies.

To show that Z_t is informationally closed with respect to Z'_t, note that the fact that H is a subgroup of G implies that Z'_t is a finer coarse-graining of X_t than Z_t, and hence there exists a coarse-graining mapping ϕ' such that $Z_t = \phi'(Z'_t)$. Then, the data processing inequality implies that

$$\begin{aligned} 0 &\le I(\bar{\boldsymbol{Z}}'_t; \vec{\boldsymbol{Z}}^L_{t+1}) - I(\bar{\boldsymbol{Z}}_t; \vec{\boldsymbol{Z}}^L_{t+1}) \\ &\le I(\bar{\boldsymbol{X}}_t; \vec{\boldsymbol{Z}}^L_{t+1}) - I(\bar{\boldsymbol{Z}}_t; \vec{\boldsymbol{Z}}^L_{t+1}) = 0. \end{aligned} \tag{S41}$$

□

Supplementary Material S5: Symmetries of Hopfield networks

Let us prove Eq. 14. For this, let $\mathscr{X} = \{-1, 1\}^n$ and let $Q = [q_1, \dots, q_m] \in \{-1, 1\}^{n\times m}$. Both learning rules considered in this work can be written as

$$W = QH, \qquad z(x) = Hx. \tag{S51}$$

For the Hebbian rule,

$$H = \frac{1}{n}Q^\top, \qquad Q^\top = nI_m H,$$

whereas for the pseudo-inverse rule, assuming $Q^\top Q$ is invertible,

$$H = (Q^\top Q)^{-1}Q^\top, \qquad Q^\top = (Q^\top Q)H.$$

Thus, in either case there exists a symmetric matrix M such that $Q^\top = MH$ with

$$M = \begin{cases} nI_m, & \text{Hebbian rule,} \\ Q^\top Q, & \text{pseudo-inverse rule.} \end{cases} \tag{S52}$$

Now, consider the group

$$G := \{g \in \mathrm{Sym}(\mathscr{X}) : z(g\cdot x) = z(x)\ \forall x \in \mathscr{X}\}, \tag{S53}$$

where $\mathrm{Sym}(\mathscr{X})$ is the group of automorphisms of the set $\mathscr{X}$. The orbits of G are exactly the fibres of z: every element of G preserves each fibre, and if $z(x) = z(x')$, the transposition exchanging x and x' while fixing all other configurations belongs to G.

To show that $p(x_{t+1}|x_t)$ is equivariant under G, first note that the logistic update rule can be expressed as

$$p(x^i_{t+1}|x_t) = \frac{\exp\big[\beta x^i_{t+1}(Wx_t)_i\big]}{2\cosh\big[\beta(Wx_t)_i\big]}, \tag{S54}$$

where $(Wx_t)i$ is the i-th coordinate of Wx_t. Combining $p(x_{t+1}|x_t) = \prod_i p\big(x^i_{t+1}|x_t\big)$ with $Wx_t = Qz(x_t)$ and $Q^\top x_{t+1} = Mz(x_{t+1})$ leads to

$$\begin{aligned} p(x_{t+1}|x_t) &= \frac{\exp\big[\beta x^\top_{t+1} Qz(x_t)\big]}{\prod_{i=1}^n 2\cosh[\beta(Qz(x_t))_i]} \\ &= \frac{\exp\big[\beta z(x_{t+1})^\top Mz(x_t)\big]}{\prod_{i=1}^n 2\cosh[\beta(Qz(x_t))_i]} \\ &=: \kappa\big(z(x_{t+1}), z(x_t)\big). \end{aligned} \tag{S55}$$

Therefore, for every $g \in G$,

$$\begin{aligned} p(g\cdot x_{t+1}|g\cdot x_t) &= \kappa\big(z(g\cdot x_{t+1}), z(g\cdot x_t)\big) \\ &= \kappa\big(z(x_{t+1}), z(x_t)\big) \\ &= p(x_{t+1} \mid x_t). \end{aligned} \tag{S56}$$

Hence, G is a global symmetry of the transition kernel, and the Mattis magnetisation is (up to a relabelling of its values) the orbit map associated with G.

Supplementary Material S6: Technical details of simulations of Hopfield networks

For the numerical experiments, Hopfield networks of n=400 neurons arranged on a 20×20 grid were trained on the rotations and reflections of the letters G, J, and R, giving rise to $m = 3\times 8 = 24$ patterns. To enhance the capacity of the network to store non-orthogonal patterns, these patterns were encoded into synaptic weights using a variation of Eq. 10 known as the *pseudo-inverse learning rule* [110]. This approach yields a synaptic weight matrix W given by

$$W = \frac{1}{m} Q\Big(\frac{1}{m}Q^\top Q\Big)^{-1} Q^\top, \tag{S61}$$

where $Q = [q_1, \dots, q_m]$ is an $n\times m$ matrix holding $m < n$ target patterns $q_\mu = (q_\mu^1, \dots, q_\mu^n) \in \{-1, 1\}^n$ as columns.

For this synaptic encoding, each pattern q_μ is an eigenvector of W with eigenvalue 1, while vectors orthogonal to the span of the patterns are also eigenvectors but with zero eigenvalue. This implies that

$$W x_t = \sum_{\mu=1}^{m} (h_\mu \cdot x_t) q_\mu, \tag{S62}$$

where $(a \cdot b) = \sum_i a_i b_i$ is the dot product, and h_μ are the rows of $(Q^\top Q)^{-1} Q^\top$, which satisfy $(h_\mu \cdot\; q_\nu) = \delta_\mu^\nu$. Note that if $q_1, \dots, q_m$ are orthogonal, then $h_\mu = q_\mu/n$. Thus, in the pseudo-inverse setting, one can define a generalised Mattis magnetisation as $Z_t = (Z_t^1, \dots, Z_t^m)$ with

$$Z_t^\mu := (h_\mu \cdot X_t), \tag{S63}$$

which reduces to the original when the patterns are orthogonal. Using an analogous reasoning as in Section II, one can show this magnetisation can be retrieved via orbits of state permutations $g \in G$ satisfying

$$\big(h_\mu \cdot (g\cdot x)\big) = (h_\mu \cdot x) \quad \forall \mu = 1, \dots, m, \forall x \in \{-1, 1\}^n. \tag{S64}$$

It can be shown that the network dynamics are equivariant with respect to this symmetry (Supplementary Material Section S5), which makes this magnetisation informationally closed.

For the experiments reported in Fig. 2E, $R = 100{,}000$ trajectories of length $T = 250$ time-steps were generated, starting from a random initial state and using an inverse temperature parameter $\beta = 2$. For a coarse-grained level associated with subgroup H, let $\mathscr{C}_H$ denote the corresponding partition of the 24 patterns into subgroup orbits. The predicted coarse state at time t was defined as the one with largest Mattis magnetisation:

$$\widehat{C}_t^H = \underset{C\in\mathscr{C}_H}{\arg\max} \left| \sum_{\mu\in C} Z_t^\mu \right|. \tag{S65}$$

The prediction error at time t (displayed in Fig. 2E) is then the fraction of trajectories for which the selected coarse state at time t differs from the selected state at the end of the run:

$$\mathrm{Err}_H(t) = \frac{1}{R} \sum_{r=1}^{R} \mathbb{1}\left\{ \widehat{C}_{r,t}^H \neq \widehat{C}_{r,250}^H \right\}. \tag{S66}$$

The control curves in Fig. 2E were calculated by replacing the symmetry-induced orbit partition at each hierarchy level with a random partition having the same number and sizes of cells — a new random partition was sampled independently for every trajectory and hierarchy level.

Supplementary Material S7: Proof of Theorem 2

Proof. Using Eq. 19a one can show that

$$p\big(z_{t+1}|g\cdot x_t\big) = \sum_{x_{t+1}\in\phi_G^{-1}(z_{t+1})} p\big(x_{t+1}|g\cdot x_t\big) \tag{S71}$$

$$= \sum_{x_{t+1}\in\phi_G^{-1}(z_{t+1})} p\big(x_{t+1}|x_t\big) \tag{S72}$$

$$= p\big(z_{t+1}|x_t\big). \tag{S73}$$

A similar calculation using Eq. 19b leads to

$$p\big(v_t|g\cdot x_t\big) = p\big(v_t|x_t\big). \tag{S74}$$

These results imply that $p\big(z_{t+1}|x_t\big) = p\big(z_{t+1}|z_t\big)$ and $p\big(v_t|x_t\big) = p\big(v_t|z_t\big)$, which imply that

$$p(\vec{\boldsymbol{z}}_0^{t+2}, \vec{\boldsymbol{v}}_0^{t+1}) = \sum_{\substack{\vec{\boldsymbol{x}}_0^{t+2}\in\phi_G^{-1}(\vec{\boldsymbol{z}}_0^{t+2})\\ \vec{\boldsymbol{y}}_0^{t+1}\in\psi_G^{-1}(\vec{\boldsymbol{v}}_0^{t+1})}} p(x_0) \prod_{\tau=0}^{t} p(x_{\tau+1}|x_\tau) p(y_\tau|x_\tau)$$

$$= \sum_{\substack{\vec{\boldsymbol{x}}_0^{t+1}\in\phi_G^{-1}(\vec{\boldsymbol{z}}_0^{t+1})\\ \vec{\boldsymbol{y}}_0^{t}\in\psi_G^{-1}(\vec{\boldsymbol{v}}_0^{t})}} p(z_{t+1}|x_t) p(v_t|x_t) p(x_0) \prod_{\tau=0}^{t-1} p(x_{\tau+1}|x_\tau) p(y_\tau|x_\tau)$$

$$= p(z_{t+1}|z_t) p(v_t|z_t) \sum_{\substack{\vec{\boldsymbol{x}}_0^{t+1}\in\phi_G^{-1}(\vec{\boldsymbol{z}}_0^{t+1})\\ \vec{\boldsymbol{y}}_0^{t}\in\psi_G^{-1}(\vec{\boldsymbol{v}}_0^{t})}} p(x_0) \prod_{\tau=0}^{t-1} p(x_{\tau+1}|x_\tau) p(y_\tau|x_\tau)$$

$$= \dots$$

$$= \prod_{\tau=0}^{t} p(z_{\tau+1}|z_\tau) p(v_\tau|z_\tau) \sum_{x_0\in\phi_G^{-1}(z_0)} p(x_0) \tag{S75}$$

$$= p(z_0) \prod_{\tau=0}^{t} p(z_{\tau+1}|z_\tau) p(v_\tau|z_\tau). \tag{S76}$$

□

Supplementary Material S8: Proof of Cor. 3

Proof. The beliefs b_t^* satisfy $b_t^* = U_{z,v}(v_t, b_{t-1}^*)$ because, thanks to Theorem 2, (Z_t, V_t) is an HMM. Furthermore,

$$b_t^* = \sum_{\tilde{\boldsymbol{y}}_t \in \psi_G^{-1}(\tilde{\boldsymbol{v}}_t)} p(\tilde{\boldsymbol{y}}_t|\tilde{\boldsymbol{v}}_t)p(z_t|\tilde{\boldsymbol{y}}_t) = \mathbb{E}\{B_t'|\overleftarrow{\boldsymbol{V}}_t = \tilde{\boldsymbol{v}}_t\}, \quad \text{(S81)}$$

with $B_t' = \mathbb{E}\{\mathbb{1}_{Z_t}|\overleftarrow{\boldsymbol{Y}}_t\} = P_{\phi_G} B_t$. □

Supplementary Material S9: Proof of Cor. 4

Proof. Eq. 26 is equivalent to

$$\mathbb{E}\left\{\log \frac{p(Z_t|\overleftarrow{\boldsymbol{Y}}_t)}{p(Z_t|\overleftarrow{\boldsymbol{V}}_t)}\right\} = 0, \quad \text{(S91)}$$

which implies that $p(z_t|\tilde{\boldsymbol{v}}_t) = p(z_t|\tilde{\boldsymbol{y}}_t)$ for all $\psi_G(\tilde{\boldsymbol{y}}_t) = \tilde{\boldsymbol{v}}_t$ almost surely. Using Eq. S81, this leads to

$$b_t^* = p(z_t|\tilde{\boldsymbol{y}}_t) \sum_{\tilde{\boldsymbol{y}}_t \in \psi_G^{-1}(\tilde{\boldsymbol{v}}_t)} p(\tilde{\boldsymbol{y}}_t|\tilde{\boldsymbol{v}}_t) = P_{\phi_G} b_t. \quad \text{(S92)}$$

□

Supplementary Material S10: Epidemic simulations

This appendix describes various technical aspects related to the analysis presented in Section II.

1. Simulation parameters

All simulations used the following parameters: $\gamma = 0.4$ corresponding to a quick recovery rate, false negative rate $\alpha = 0.1$, and false positive rate $\delta = 0.05$.

For the analysis reported in Fig. 7C, we considered populations composed of $m = 2$ communities of equal sizes ranging from 2 up to 50, and used $\beta_{\ell,\ell} = 0.06$ for intra-community and $\beta_{\ell,\ell'} = 0.01$ for inter-community per-contact transmission rate. The reported runtime is the mean of 50 consecutive filtering updates. Transition- and emission-table precomputation for the exact abstract filter was timed separately and excluded from the reported per-update runtime. The mean-field curve in Fig. 7C corresponds to the scalar aggregate mean-field.

The analysis reported in Fig. 7D considers two communities of sizes $n_1 = 120$ and $n_2 = 40$. The intra-community transmission rates were established via $\beta_{\ell,\ell} = R_0^{(\ell)}\gamma/n_\ell$. To account for heterogeneous susceptibilities, $R_0^{(1)} = 1.2$ and $R_0^{(2)} = 8.0$ were used. Inter-community transmission rates were defined as

$$\beta_{i,j} = \frac{\min_\ell \beta_{\ell,\ell}}{2} \quad \text{for all } i,j \in \{1,\dots,m\}, i \neq j. \quad \text{(S101)}$$

A single trajectory of $T = 40$ timepoints was generated; at $t = 0$, infections were sampled independently for every individual with probability 0.3. Belief updating for abstract beliefs was initialised uniformly over the $121 \times 41 = 4961$ possible infection counts for both communities. The per-individual mean-field filter was initialised with infection probability $\theta_i = 0.5$ for every individual. The scalar aggregate mean-field filter was initialised with mean $\mu_0 = (n_1 + n_2)/2 = 80$ and variance $P_0 = (n_1 + n_2)/4 = 40$. The bars in Fig. 7D summarise performance over the 40 timepoints of this single trajectory, rather than over multiple independently simulated trajectories.

2. Why the total infection count can be non-Markovian

Let us denote by $W_t = \sum_{\ell=1}^m Z_t^\ell$ the total number of infections at time t. Thanks to our results, it is clear that $Z_t = (Z_t^1, \dots, Z_t^m)$ has Markovian dynamics. However, note that this does not imply that the sum of its components is also Markovian. In general, if the dynamics of the various components of Z_t are very different (due to distinct sizes and/or infection rates), this enhances the degree of non-Markovianity of W_t. For this reason, any estimation technique that assumes Markovianity would necessarily make errors.

3. Construction of mean-field approximations

Aggregate mean-field filter

The aggregate filter collapses the community structure entirely, and tracks only the total infection count $W_t = \sum_i X_t^i$ via a scalar Gaussian state (μ_t, P_t), where

$$\mu_t = \mathbb{E}\big[W_t\big|\vec{\boldsymbol{V}}_0^{t+1}\big] \quad \text{and} \quad P_t = \mathrm{Var}\big[W_t\big|\vec{\boldsymbol{V}}_0^{t+1}\big]. \quad \text{(S102)}$$

For building this approximation, the community-dependent hazards are replaced by a single population-weighted effective rate

$$\beta_{\text{eff}} = \sum_{\ell,r=1}^m \beta_{\ell,r}\frac{n_\ell n_r}{n^2}, \quad \text{(S103)}$$

which represents the average per-capita transmission rate experienced by a uniformly drawn individual. Setting $p_{\text{inf}} = 1 - e^{-\beta_{\text{eff}}\,\mu_t}$, the mean and variance are propagated via moment closure:

$$\mu_{t+1|t} = (1-\gamma)\,\mu_t + (n-\mu_t)\,p_{\text{inf}}, \quad \text{(S104)}$$
$$P_{t+1|t} = (f')^2\,P_t + Q, \quad \text{(S105)}$$

where $f' = (1-\gamma) - p_{\text{inf}} + (n-\mu_t)\,\beta_{\text{eff}}\,e^{-\beta_{\text{eff}}\,\mu_t}$ is the Jacobian of the mean-update and $Q = \mu_t\gamma(1-\gamma) + (n-\mu_t)\,p_{\text{inf}}(1-p_{\text{inf}})$ captures the binomial process noise.

The total observation $V_t^{\text{total}} = \sum_\ell V_t^\ell$ serves as a scalar measurement with sensitivity $H = 1 - \alpha - \delta$ and observation noise $R = n\,\mathbb{E}[Y_i](1 - \mathbb{E}[Y_i])$. Then, the standard Kalman equations give

$$S = H^2 P_{t+1|t} + R, \tag{S106}$$
$$K = P_{t+1|t}\, H/S, \tag{S107}$$
$$\mu_{t+1} = \mu_{t+1|t} + K\,(V_{t+1}^{\text{total}} - n\,\mathbb{E}[Y_i]), \tag{S108}$$
$$P_{t+1} = (1 - KH)\, P_{t+1|t}. \tag{S109}$$

The cost per step is $O(1)$, independent of both n and m.

Although this filter is computationally attractive, it incurs a structural bias whenever communities have heterogeneous transmission rates. The root cause is that W_t is *not* a Markov chain in such settings: its one-step distribution depends not only on the current total but on how those infections are distributed across communities — because communities with different β_{ll} and n_l drive the contagion at different rates. Two configurations with the same total count but different community breakdowns will therefore evolve differently, so no function of W_t alone can capture all the dynamically relevant information. This aggregate filter ignores this dependence by treating W_t as Markovian with dynamics governed by the single effective rate β_{eff} defined in Eq. S103, which is exact only when all communities are statistically identical.

Per-individual mean-field filter

The per-individual mean-field filter maintains, for each individual i, the marginal probability $\theta_t^i := \mathbb{P}(X_t^i = 1 \mid \vec{\boldsymbol{V}}_0^{t+1})$. The n-dimensional vector $\theta_t = (\theta_t^1, \ldots, \theta_t^n)$ plays the role of a factorised belief: all individual states are treated as conditionally independent given the observations. The filter alternates between a prediction step and an extended Kalman-style correction.

Under the mean-field approximation, community infection counts are replaced by their expected values $\bar{z}_t^l = \sum_{i:\, c_i = l} \theta_t^i$, giving effective hazards $h_l(\bar{z}_t)$ and per-individual infection probabilities $\bar{p}_{\text{inf}}^i = 1 - e^{-h_{c_i}(\bar{z}_t)}$. Each marginal is then propagated forward via

$$\theta_{t+1|t}^i = (1 - \theta_t^i)\, \bar{p}_{\text{inf}}^i + \theta_t^i\,(1 - \gamma), \tag{S1010}$$

which mirrors the SIS dynamics at the level of individual marginals.

The aggregate community observation $V_t^l = \sum_{i:\, c_i = l} Y_t^i$ is used to correct the predicted marginals. Under the mean-field independence assumption the moments of V_t^l are

$$\mathbb{E}\big[V_t^l\big] = \sum_{i:\, c_i = l} \big(\delta + (1 - \alpha - \delta)\,\theta_{t|t-1}^i\big), \tag{S1011}$$
$$\mathrm{Var}\big[V_t^l\big] = \sum_{i:\, c_i = l} \mathrm{Var}[Y_t^i], \tag{S1012}$$

and the covariance between individual i and the aggregate is $\mathrm{Cov}(X_{t+1}^i, V_{t+1}^l) = (1 - \alpha - \delta)\,\theta_{t+1|t}^i(1 - \theta_{t+1|t}^i)$. This yields the individual Kalman gain $K_i = \mathrm{Cov}(X_{t+1}^i, V_{t+1}^l)/\mathrm{Var}[V_{t+1}^l]$, and the posterior marginals are updated as

$$\theta_{t+1}^i = \theta_{t+1|t}^i + K_i\,\big(V_{t+1}^l - \mathbb{E}[V_{t+1}^l]\big). \tag{S1013}$$

The posterior mean and variance of Z_{t+1}^l are then $\sum_{i:\, c_i = l} \theta_{t+1}^i$ and $\sum_{i:\, c_i = l} \theta_{t+1}^i(1 - \theta_{t+1}^i)$, respectively. The cost per step is $O(n)$, linear in population size.

In contrast with the aggregate mean-field filter, the per-individual mean-field filter achieves good performance in terms of mean value. However, because it ignores interactions between individuals, this filter makes its posterior substantially wider than the optimal posterior. Thus, the per-individual mean-field filter provides a good prediction but underestimates the resulting confidence one should have in it.

Supplementary Material S11: Parallels with renormalisation group theory

Here we discuss the similarities and differences between renormalisation group (RG) theory and the framework presented in this paper. Both approaches are attempts to explain why different levels of description of a system can have autonomous laws. Moreover, they both do this by constructing simplified macroscopic descriptions that disregard microscopic degrees of freedom. However, they have important differences highlighted below.

For concreteness, consider a real-space renormalisation transformation of a finite statistical-mechanical system [9, 10]. Let $\mathscr{X}$ denote a microscopic configuration space, and let $H : \mathscr{X} \to \mathbb{R}$ be a dimensionless Hamiltonian, where the inverse temperature has been absorbed into H. A blocking transformation can be represented by a stochastic kernel

$$W(z|x) \geq 0, \qquad \sum_{z \in \mathscr{Z}} W(z|x) = 1. \tag{S111}$$

A deterministic coarse-graining $\phi : \mathscr{X} \to \mathscr{Z}$ is recovered as the special case $W(z|x) = \delta_{z,\phi(x)}$. A block Hamiltonian $H_b : \mathscr{Z} \to \mathbb{R}$ is defined by

$$e^{-H_b(z)} = \sum_{x \in \mathscr{X}} W(z|x) e^{-H(x)}. \tag{S112}$$

This blocking step, $\mathscr{B}(H) = H_b$, changes the resolution and, for a finite system, the number of degrees of freedom. A complete RG step, therefore, also includes a rescaling of coordinates and fields that identifies the blocked theory with a theory expressed on the original phase space. By denoting the rescaling by $\mathscr{S}$, a complete RG step is expressed as

$$\mathbf{RG}(H) = \mathscr{S}\left[\mathscr{B}(H)\right]. \tag{S113}$$

Thus, **RG** can be regarded as a self-mapping of a space of Hamiltonians. Iterations of this mapping generate an RG flow $\mathbf{RG}(H^k) = H^{k+1}$. Eq. S112 can be exact; approximation typically enters when the resulting Hamiltonian is restricted to a tractable family of operators or couplings.

1. Different roles of symmetry

Symmetry enters RG by constraining the blocking transformation and the resulting effective theory. Suppose that a group G acts on both $\mathscr{X}$ and $\mathscr{Z}$. A symmetry-preserving blocking kernel satisfies

$$W(g\cdot z|g\cdot x) = W(z|x), \quad \forall\, x\in\mathscr{X},\ z\in\mathscr{Z},\ g\in G. \tag{S114}$$

If the microscopic Hamiltonian is invariant, $H(g\cdot x) = H(x)$, then the renormalised Hamiltonian can be shown to inherit the same symmetry:

$$\begin{aligned} e^{-H_b(g\cdot z)} &= \sum_{x\in\mathscr{X}} W(g\cdot z|x)e^{-H(x)} \\ &= \sum_{\tilde{x}\in\mathscr{X}} W(g\cdot z|g\cdot\tilde{x})e^{-H(g\cdot\tilde{x})} \\ &= \sum_{\tilde{x}\in\mathscr{X}} W(z|\tilde{x})e^{-H(\tilde{x})} = e^{-H_b(z)}. \end{aligned} \tag{S115}$$

Thus, in this setting symmetries constrain the renormalisation transformation and the operators that may appear in the resulting effective theory. If rescaling $\mathscr{S}$ is also compatible with the action of G, the complete RG transformation maps G-invariant theories into G-invariant theories. Near an RG fixed point, symmetry also organises perturbations into representations of G, while the eigenvalues of the linearised RG transformation determine whether these perturbations are relevant, marginal, or irrelevant.

Importantly, Eq. S114 does not determine W. Majority-rule transformations, decimation, and stochastic blocking transformations can preserve the same physical symmetry while retaining different variables. Locality, scale, the observables of interest, or an optimisation criterion must additionally be used to select the transformation [65, 66]. Symmetry therefore restricts the admissible RG transformations, but does not generally specify their fibres.

The framework presented in this paper uses symmetry differently. Let X_t be a Markov process on $\mathscr{X}$ whose transition probabilities satisfy

$$p(g\cdot x_{t+1}|g\cdot x_t) = p(x_{t+1}|x_t), \quad \forall x_t, x_{t+1}\in\mathscr{X},\ g\in G. \tag{S116}$$

The associated coarse-graining is not an independently selected equivariant mapping. It is the orbit mapping

$$\phi_G : \mathscr{X} \longrightarrow \mathscr{X}/G, \qquad \phi_G(x) = O_x, \tag{S117}$$

which satisfies the invariance condition

$$\phi_G(g\cdot x) = \phi_G(x), \qquad \forall x\in\mathscr{X},\ g\in G. \tag{S118}$$

Consequently, the chosen action of G is factored out rather than retained as a non-trivial action on the quotient variable.

2. Axis of reduction: scale versus symmetries

Both approaches remove information, and hence their application is non-invertible. However, the type of information that each approach removes is quite different.

RG organises detail by scale. Accordingly, coarse-grainings in RG are geometric and local, reducing neighbouring sites (following a given notion of neighbourhood). Thus, by heuristically choosing coarse-grainings based on spatial considerations, it removes short-wavelength structure while keeping large-scale structure.

In contrast, the presented framework organises information following the given symmetries: it removes the information pertaining to the specific representative within the orbit, and keeps the orbit label. Thus, the coarse-graining identifies states that may not be spatially local or at the same scale at all — it can involve a permutation of two distant particles, or reflect a whole letter pattern.

3. Scaling of perturbations versus exact dynamical closure

The two frameworks use different criteria to determine which microscopic details may be disregarded.

In RG, the blocking transformation itself can be exact: integrating out microscopic degrees of freedom transfers their effects into the interactions of the blocked Hamiltonian through Eq. S112. The distinction between relevant and irrelevant contributions concerns how perturbations of a fixed point change under repeated blocking and rescaling.

To see this, consider a Hamiltonian close to an RG fixed point H^*, written as

$$H = H^* + \sum_i u_i\mathscr{O}_i, \tag{S119}$$

where $\mathscr{O}_i$ are eigenoperators of the linearised RG transformation. To leading order, the couplings transform as

$$u_i' = b^{\xi_i}u_i + O(u^2), \tag{S1110}$$

where $b > 1$ is the scale factor and $\xi_i \in \mathbb{R}$. Perturbations with $\xi_i > 0$ are relevant, those with $\xi_i < 0$ are irrelevant, and those with $\xi_i = 0$ are marginal. An irrelevant perturbation need not have a small or vanishing effect at a finite scale; rather, its relative influence decreases under repeated RG transformations. Approximation enters when the effective Hamiltonian is truncated by neglecting such operators, rather than from the exact blocking operation itself.

The criterion employed in the present framework is different. For a coarse-graining $Z_t = \phi(X_t)$, the informational closure discrepancy is

$$\Delta_\phi^{(L)} := I\big(\overleftarrow{\boldsymbol{X}}_t; \vec{\boldsymbol{Z}}_{t+1}^L\big) - I\big(\overleftarrow{\boldsymbol{Z}}_t; \vec{\boldsymbol{Z}}_{t+1}^L\big) \geq 0. \qquad \text{(S1111)}$$

Informational closure corresponds to $\Delta_\phi^{(L)} = 0$ for all $L \in \mathbb{N}$. Consequently, once the coarse-grained past is known, microscopic information provides no additional information about the future coarse-grained process.

This exact conditional independence should not be interpreted as stating that within-orbit information is irrelevant. The microscopic representative may have non-trivial dynamical consequences, and may be essential for predicting microscopic observables. Information closure states that microscopic details are redundant only with respect to predicting macroscopic future events. Thus, while RG relevance quantifies how the influence of a perturbation grows or decays across scales, informational closure determines whether microscopic distinctions provide any additional predictive information at a given level of description.

The two criteria are, therefore, complementary rather than mutually exclusive. RG can determine whether discrepancies from a symmetric effective theory grow or decay under changes of scale; informational closure determines whether the resulting orbit variables possess autonomous dynamics.

REFERENCES

1. D. H. Wolpert, The lack of a priori distinctions between learning algorithms. *Neural Comput.* **8**, 1341–1390 (1996).

2. S. P. Adam, S.-A. N. Alexandropoulos, P. M. Pardalos, M. N. Vrahatis, “No free lunch theorem: A review,” in *Approximation and Optimization: Algorithms, Complexity and Applications* (Springer, 2019), pp. 57–82.

3. S. C. Gibb, R. F. Hendry, T. Lancaster, Eds. *The Routledge Handbook of Emergence* (Routledge, 2019).

4. S. M. Carroll, A. Parola, “What emergence can possibly mean,” in *Dennett’s Real Patterns in Science and Nature*, T. Millhouse, S. Petersen, D. Ross, Eds. (The MIT Press, 2026), pp. 113–136.

5. D. C. Krakauer, Symmetry–simplicity, broken symmetry–complexity. *Interface Focus* **13**, 20220075 (2023).

6. F. E. Rosas, B. C. Geiger, A. I. Luppi, A. K. Seth, D. Polani, M. Gastpar, P. A. M. Mediano, Software in the natural world: A computational approach to hierarchical emergence. arXiv:2402.09090 [nlin.AO] (2024).

7. D. C. Krakauer, M. Mitchell, J. W. Krakauer, Large language models and emergence: A complex systems perspective. *Philos. Trans. A Math. Phys. Eng. Sci.* **384**, 20250014 (2026).

8. P. W. Anderson, More is different: Broken symmetry and the nature of the hierarchical structure of science. *Science* **177**, 393–396 (1972).

9. L. P. Kadanoff, Scaling laws for Ising models near Tc. *Phys. Phys. Fiz.* **2**, 263 (1966).

10. K. G. Wilson, Problems in physics with many scales of length. *Sci. Am.* **241**, 158–179 (1979).

11. R. V. Solé, *Phase Transitions* (Princeton Univ. Press, 2011), vol. 16.

12. N. Dupuis, L. Canet, A. Eichhorn, W. Metzner, J. M. Pawlowski, M. Tissier, N. Wschebor, The nonperturbative functional renormalization group and its applications. *Phys. Rep.* **910**, 1–114 (2021).

13. A. K. Seth, Measuring autonomy and emergence via Granger causality. *Artif. Life* **16**, 179–196 (2010).

14. E. P. Hoel, L. Albantakis, G. Tononi, Quantifying causal emergence shows that macro can beat micro. *Proc. Natl. Acad. Sci. U.S.A.* **110**, 19790–19795 (2013).

15. F. E. Rosas, P. A. M. Mediano, H. J. Jensen, A. K. Seth, A. B. Barrett, R. L. Carhart-Harris, D. Bor, Reconciling emergences: An information-theoretic approach to identify causal emergence in multivariate data. *PLOS Comput. Biol.* **16**, e1008289 (2020).

16. L. Barnett, A. K. Seth, Dynamical independence: Discovering emergent macroscopic processes in complex dynamical systems. *Phys. Rev. E* **108**, 014304 (2023).

17. M. A. Bedau, P. Humphreys, Eds. *Emergence: Contemporary Readings in Philosophy and Science* (MIT Press, 2008).

18. P. Clayton, P. Davies, Eds. *The Re-emergence of Emergence: The Emergentist Hypothesis from Science to Religion* (Oxford Univ. Press, 2006).

19. H. J. Jensen, *Complexity Science: The Study of Emergence* (Cambridge Univ. Press, 2022).

20. J. P. Crutchfield, The calculi of emergence: Computation, dynamics and induction. *Phys. D Nonlinear Phenom.* **75**, 11–54 (1994).

21. N. Israeli, N. Goldenfeld, Coarse-graining of cellular automata, emergence, and the predictability of complex systems. *Phys. Rev. E* **73**, 026203 (2006).

22. D. Krakauer, N. Bertschinger, E. Olbrich, J. C. Flack, N. Ay, The information theory of individuality. *Theory Biosci.* **139**, 209–223 (2020).

23. C. P. Kempes, D. C. Krakauer, The multiple paths to multiple life. *J. Mol. Evol.* **89**, 415–426 (2021).

24. N. Bertschinger, E. Olbrich, N. Ay, J. Jost, "Information and closure in systems theory," in *Explorations in the Complexity of Possible Life. Proceedings of the 7th German Workshop of Artificial Life* (IOS Press, 2006), pp. 9–21.

25. A. Y. C. Chang, M. Biehl, Y. Yen, R. Kanai, Information closure theory of consciousness. *Front. Psychol.* **11**, 1504 (2020).

26. K. H. G. Vollbrecht, R. F. Werner, Entanglement measures under symmetry. *Phys. Rev. A* **64**, 062307 (2001).

27. A. Kolchinsky, D. H. Wolpert, Work, entropy production, and thermodynamics of information under protocol constraints. *Phys. Rev. X* **11**, 041024 (2021).

28. O. Reynolds, On the dynamical theory of incompressible viscous fluids and the determination of the criterion. *Phil. Trans. R. Soc. A* **186**, 123–164 (1895).

29. M. J. Field, Equivariant dynamical systems. *Trans. Am. Math. Soc.* **259**, 185–205 (1980).

30. M. Golubitsky, I. Stewart, D. G. Schaeffer, *Singularities and Groups in Bifurcation Theory: Volume II*, volume 69 (Springer Science & Business Media, 2012).

31. H. A. Makse, P. Boldi, F. Sorrentino, I. Stewart, Symmetries of living systems: Symmetry fibrations and synchronization in biological networks. arXiv:2502.18713 [q-bio.MN] (2025).

32. J. G. Kemeny, J. L. Snell, *Finite Markov Chains* (van Nostrand Princeton, 1969).

33. S.-I. Amari, Learning patterns and pattern sequences by self-organizing nets of threshold elements. *IEEE Trans. Comput.* **11**, 1197–1206 (1972).

34. J. J. Hopfield, Neural networks and physical systems with emergent collective computational abilities. *Proc. Natl. Acad. Sci. U.S.A.* **79**, 2554–2558 (1982).

35. G. E. Hinton, T. J. Sejnowski, "Learning and relearning in Boltzmann machines," in *Parallel Distributed Processing: Explorations in the Microstructure of Cognition*, *Volume 1: Foundations*, D. E. Rumelhart, J. L. McClelland, the PDP Research Group, Eds. (MIT Press, 1986), chap. 7, pp. 282–317.

36. D. Krotov, A new frontier for Hopfield networks. *Nat. Rev. Phys.* **5**, 366–367 (2023).

37. M. Aguilera, P. A. Morales, F. E. Rosas, H. Shimazaki, Explosive neural networks via higher-order interactions in curved statistical manifolds. *Nat. Commun.* **16**, 6511 (2025).

38. D. J. Amit, *Modeling Brain Function: The World of Attractor Neural Networks* (Cambridge Univ. Press, 1989).

39. W. Gerstner, W. M. Kistler, R. Naud, L. Paninski, *Neuronal Dynamics: From Single Neurons to Networks and Models of Cognition* (Cambridge Univ. Press, 2014).

40. D. O. Hebb, *The Organization of Behavior* (Wiley & Sons, 1949).

41. A. Barra, M. Beccaria, A. Fachechi, A new mechanical approach to handle generalized Hopfield neural networks. *Neural Netw.* **106**, 205–222 (2018).

42. E. Agliari, A. Alessandrelli, A. Barra, F. Ricci-Tersenghi, Parallel learning by multitasking neural networks. *J. Stat. Mech. Theory Exp.* **2023**, 113401 (2023).

43. S. A. Kauffman, Metabolic stability and epigenesis in randomly constructed genetic nets. *J. Theor. Biol.* **22**, 437–467 (1969).

44. S. Bornholdt, Boolean network models of cellular regulation: Prospects and limitations. *J. R. Soc. Interface* **5**, S85 (2008).

45. A. Saadatpour, R. Albert, Boolean modeling of biological regulatory networks: A methodology tutorial. *Methods* **62**, 3–12 (2013).

46. W. Abou-Jaoudé, P. Traynard, P. T. Monteiro, J. Saez-Rodriguez, T. Helikar, D. Thieffry, C. Chaouiya, Logical modeling and dynamical analysis of cellular networks. *Front. Genet.* **7**, 94 (2016).

47. I. Shmulevich, E. R. Dougherty, W. Zhang, Gene perturbation and intervention in probabilistic Boolean networks. *Bioinformatics* **18**, 1319–1331 (2002).

48. A. Jenkins, M. Macauley, Bistability and asynchrony in a Boolean model of the L-arabinose operon in *Escherichia coli*. *Bull. Math. Biol.* **79**, 1778–1795 (2017).

49. S. Särkkä, L. Svensson, *Bayesian Filtering and Smoothing* (Cambridge Univ. Press, 2023), vol. 17.

50. P. Ehrenfest, T. Ehrenfest-Afanassjewa, Über zwei bekannte Einwände gegen das Boltzmannsche H-Theorem. *Phys. Z.* **8**, 311–314 (1907).

51. H. W. Hethcote, The mathematics of infectious diseases. *Siam Rev.* **42**, 599–653 (2000).

52. R. Pastor-Satorras, C. Castellano, P. Van Mieghem, A. Vespignani, Epidemic processes in complex networks. *Rev. Mod. Phys.* **87**, 925–979 (2015).

53. L. J. S. Allen, "An introduction to stochastic epidemic models," in *Mathematical Epidemiology* (Springer, 2008), pp. 81–130.

54. R. Brookmeyer, D. F. Stroup, *Monitoring the Health of Populations: Statistical Principles and Methods for Public Health Surveillance* (Oxford Univ. Press, 2004).

55. E. Hoel, Quantifying emergent complexity. *Patterns* **7**, 101472 (2026).

56. A. Jansma, E. Hoel, Engineering emergence. arXiv:2510.02649 [cs.IT] (2025).

57. T. F. Varley, Flickering emergences: The question of locality in information-theoretic approaches to emergence. *Entropy* **25**, 54 (2023).

58. H. M. Tolle, A. I. Luppi, A. K. Seth, P. A. M. Mediano, Evolving reservoir computers reveal bidirectional coupling between predictive power and emergent dynamics. *Patterns* **7**, 101457 (2026).

59. F. Pigozzi, A. Goldstein, M. Levin, Associative conditioning in gene regulatory network models increases integrative causal emergence. *Commun. Biol.* **8**, 1027 (2025).

60. P. W. Anderson, "Some general thoughts about broken symmetry," in *A Career in Theoretical Physics* (World Scientific, 1994), pp. 419–429.

61. M. Fruchart, R. Hanai, P. B. Littlewood, V. Vitelli, Non-reciprocal phase transitions. *Nature* **592**, 363–369 (2021).

62. R. G. Palmer, Broken ergodicity. *Adv. Phys.* **31**, 669–735 (1982).

63. R. W. Batterman, *The Devil in the Details: Asymptotic Reasoning in Explanation, Reduction, and Emergence* (Oxford Univ. Press, 2001).

64. D. S. Berman, M. S. Klinger, A. G. Stapleton, Bayesian renormalization. *Mach. Learn. Sci. Technol.* **4**, 045011 (2023).

65. M. Koch-Janusz, Z. Ringel, Mutual information, neural networks and the renormalization group. *Nat. Phys.* **14**, 578–582 (2018).

66. P. M. Lenggenhager, D. E. Gökmen, Z. Ringel, S. D. Huber, M. Koch-Janusz, Optimal renormalization group transformation from information theory. *Phys. Rev. X* **10**, 011037 (2020).

67. K. Friston, C. Heins, T. Verbelen, L. Da Costa, T. Salvatori, D. Markovic, A. Tschantz, M. Koudahl, C. Buckley, T. Parr, From pixels to planning: Scale-free active inference. *Front. Netw. Physiol.* **5**, 1521963 (2025).

68. C. Fefferman, S. Mitter, H. Narayanan, Testing the manifold hypothesis. *J. Am. Math. Soc.* **29**, 983–1049 (2016).

69. V. Thibeault, A. Allard, P. Desrosiers, The low-rank hypothesis of complex systems. *Nat. Phys.* **20**, 294–302 (2024).

70. D. McSharry, C. Kaplanis, F. E. Rosas, P. A. Mediano, Learning diverse causally emergent representations from time series data. *Adv. Neural Inf. Proces. Syst.* **37**, 119547–119572 (2024).

71. H. Charvin, N. C. Volpi, D. Polani, Towards information theory-based discovery of equivariances. arXiv:2310.16555 [cs.IT] (2023).

72. F. E. Rosas, Adaptive state-action abstractions via rate-distortion. arXiv:2606.06123 [cs.LG] (2026).

73. M. M. Bronstein, J. Bruna, T. Cohen, P. Veličković, Geometric deep learning: Grids, groups, graphs, geodesics, and gauges. arXiv:2104.13478 [cs.LG] (2021).

74. L.-H. Lim, B. J. Nelson, What is an equivariant neural network? *Not. Am. Math. Soc.* **70**, 619–625 (2023).

75. R. C. Conant, W. R. Ashby, Every good regulator of a system must be a model of that system. *Int. J. Syst. Sci.* **1**, 89–97 (1970).

76. W. R. Ashby, J. Goldstein, Variety, constraint, and the law of requisite variety. *Emergence: Complexity and Organization* **13**, 190 (2011).

77. N. Virgo, M. Biehl, M. Baltieri, M. Capucci, A "good regulator theorem" for embodied agents. arXiv:2508.06326 [cs.AI] (2025).

78. M. Baltieri, M. Biehl, M. Capucci, N. Virgo, A Bayesian interpretation of the internal model principle. arXiv:2503.00511 [math.OC] (2025).

79. M. K. Ho, D. Abel, T. L. Griffiths, M. L. Littman, The value of abstraction. *Curr. Opin. Behav. Sci.* **29**, 111–116 (2019).

80. M. K. Ho, D. Abel, C. G. Correa, M. L. Littman, J. D. Cohen, T. L. Griffiths, People construct simplified mental representations to plan. *Nature* **606**, 129–136 (2022).

81. B. De Martino, A. Cortese, Goals, usefulness and abstraction in value-based choice. *Trends Cogn. Sci.* **27**, 65–80 (2023).

82. F. Chollet, On the measure of intelligence. arXiv:1911.01547 [cs.AI] (2019).

83. A. Geiger, D. Ibeling, A. Zur, M. Chaudhary, S. Chauhan, J. Huang, A. Arora, W. Zhengxuan, N. Goodman, C. Potts, T. Icard, Causal abstraction: A theoretical foundation for mechanistic interpretability. *J. Mach. Learn. Res.* **26**, 1–64 (2025).

84. G. Konidaris, On the necessity of abstraction. *Curr. Opin. Behav. Sci.* **29**, 1–7 (2019).

85. D. Abel, "A theory of abstraction in reinforcement learning," thesis, Brown University (2020).

86. C. Allen, "Structured abstractions for general-purpose decision making," thesis, Brown University (2023).

87. E. Van der Pol, D. Worrall, H. van Hoof, F. Oliehoek, M. Welling, MDP homomorphic networks: Group symmetries in reinforcement learning. *Adv. Neural Inf. Proces. Syst.* **33**, 4199–4210 (2020).

88. L. P. Kaelbling, M. L. Littman, A. R. Cassandra, Planning and acting in partially observable stochastic domains. *Artif. Intell.* **101**, 99–134 (1998).

89. A. Di Bernardo, A. Valente, F. Mastrogiuseppe, S. Ostojic, Shaping Slow Manifolds in Equivariant Recurrent Neural Networks. *Neural Comput.* **38**, 1913–1981 (2026).

90. T. A. Keller, Flow equivariant recurrent neural networks. arXiv:2507.14793 [cs.LG] (2025).

91. P. Buchholz, Exact and ordinary lumpability in finite Markov chains. *J. Appl. Prob.* **31**, 59–75 (1994).

92. B. C. Geiger, T. Petrov, G. Kubin, H. Koeppl, Optimal Kullback–Leibler aggregation via information bottleneck. *IEEE Trans. Automat. Contr.* **60**, 1010–1022 (2015).

93. B. C. Geiger, C. Temmel, Lumpings of Markov chains, entropy rate preservation, and higher-order lumpability. *J. Appl. Probab.* **51**, 1114–1132 (2014).

94. M. Faccin, M. T. Schaub, J.-C. Delvenne, State aggregations in Markov chains and block models of networks. *Phys. Rev. Lett.* **127**, 078301 (2021).

95. A. C. Costa, T. Ahamed, D. Jordan, G. J. Stephens, Maximally predictive states: From partial observations to long timescales. *Chaos* **33**, 023136 (2023).

96. P. L. Simon, M. Taylor, I. Z. Kiss, Exact epidemic models on graphs using graph-automorphism driven lumping. *J. Math. Biol.* **62**, 479–508 (2011).

97. S. Banisch, R. Lima, Markov chain aggregation for simple agent-based models on symmetric networks: The voter model. *Adv. Complex Syst.* **18**, 1550011 (2015).

98. L. B. White, R. Mahony, G. D. Brushe, Lumpable hidden Markov models—Model reduction and reduced complexity filtering. *IEEE Trans. Automat. Contr.* **45**, 2297–2306 (2000).

99. R. M. Shiffrin, M. D. Lee, W. Kim, E.-J. Wagenmakers, A survey of model evaluation approaches with a tutorial on hierarchical Bayesian methods. *Cognit. Sci.* **32**, 1248–1284 (2008).

100. D. M. Blei, A. Kucukelbir, J. D. McAuliffe, Variational inference: A review for statisticians. *J. Am. Stat. Assoc.* **112**, 859–877 (2017).

101. D. R. Sheldon, T. Dietterich, Collective graphical models. *Adv. Neural Inf. Proces. Syst.* **24**, 9 (2011).

102. D. Sheldon, T. Sun, A. Kumar, T. Dietterich, Approximate inference in collective graphical models, in *International Conference on Machine Learning*, (PMLR, 2013), pp. 1004–1012.

103. D. Poole, "First-order probabilistic inference," in IJCAI'03: *Proceedings of the 18th International Joint Conference on Artificial intelligence* (Morgan Kaufmann Publishers Inc., 2003), pp. 985–991.

104. R. de Salvo Braz, E. Amir, D. Roth, "Lifted first-order probabilistic inference," in *Proceedings of the Nineteenth International Joint Conference on Artificial Intelligence* (Morgan Kaufmann, 2005), pp. 1319–1325.

105. B. Milch, L. S. Zettlemoyer, K. Kersting, M. Haimes, L. P. Kaelbling, "Lifted probabilistic inference with counting formulas," in *AAAI'08: Proceedings of the 23rd National Conference on Artificial intelligence* (AAAI, 2008), pp. 1062–1068.

106. M. Niepert, G. Van den Broeck, "Tractability through exchangeability: A new perspective on efficient probabilistic inference," in *AAAI'14: Proceedings of the Twenty-Eighth AAAI Conference on Artificial Intelligence* (AAAI, 2014), pp. 2467–2475.

107. S. Lüdtke, M. Schröder, F. Krüger, S. Bader, T. Kirste, State-space abstractions for probabilistic inference: A systematic review. *J. Artif. Intell. Res.* **63**, 789–848 (2018).

108. M. Schröder, S. Lüdtke, S. Bader, F. Krüger, T. Kirste, "LiMa: Sequential lifted marginal filtering on multiset state descriptions," in *Joint German/Austrian Conference on Artificial Intelligence (Künstliche Intelligenz)* (Springer, 2017), pp. 222–235.

109. M. Gehrke, T. Braun, R. Möller, "Lifted dynamic junction tree algorithm," in *International Conference on Conceptual Structures* (Springer, 2018), pp. 55–69.

110. I. Kanter, H. Sompolinsky, Associative recall of memory without errors. *Phys. Rev. A Gen. Phys.* **35**, 380 (1987).
